# Proteomic analysis of necroptotic extracellular vesicles

**DOI:** 10.1038/s41419-021-04317-z

**Published:** 2021-11-08

**Authors:** Inbar Shlomovitz, Ziv Erlich, Gali Arad, Liat Edry-Botzer, Sefi Zargarian, Hadar Cohen, Tal Manko, Yifat Ofir-Birin, Tomer Cooks, Neta Regev-Rudzki, Motti Gerlic

**Affiliations:** 1 Department of Clinical Microbiology and Immunology, Sackler School of Medicine, Tel Aviv University, Tel Aviv, Israel; 2 Department of Human Molecular Genetics and Biochemistry, Sackler School of Medicine, Tel Aviv University, Tel Aviv, Israel; 3 The Shraga Segal Department of Microbiology, Immunology and Genetics, Ben-Gurion University of the Negev, Beer-Sheva, Israel; 4 Department of Biomolecular Sciences, Weizmann Institute of Science, Rehovot, Israel

**Keywords:** Necroptosis, Cell death and immune response

## Abstract

Necroptosis is a regulated and inflammatory form of cell death. We, and others, have previously reported that necroptotic cells release extracellular vesicles (EVs). We have found that necroptotic EVs are loaded with proteins, including the phosphorylated form of the key necroptosis-executing factor, mixed lineage kinase domain-like kinase (MLKL). However, neither the exact protein composition, nor the impact, of necroptotic EVs have been delineated. To characterize their content, EVs from necroptotic and untreated U937 cells were isolated and analyzed by mass spectrometry-based proteomics. A total of 3337 proteins were identified, sharing a high degree of similarity with exosome proteome databases, and clearly distinguishing necroptotic and control EVs. A total of 352 proteins were significantly upregulated in the necroptotic EVs. Among these were MLKL and caspase-8, as validated by immunoblot. Components of the ESCRTIII machinery and inflammatory signaling were also upregulated in the necroptotic EVs, as well as currently unreported components of vesicle formation and transport, and necroptotic signaling pathways. Moreover, we found that necroptotic EVs can be phagocytosed by macrophages to modulate cytokine and chemokine secretion. Finally, we uncovered that necroptotic EVs contain tumor neoantigens, and are enriched with components of antigen processing and presentation. In summary, our study reveals a new layer of regulation during the early stage of necroptosis, mediated by the secretion of specific EVs that influences the microenvironment and may instigate innate and adaptive immune responses. This study sheds light on new potential players in necroptotic signaling and its related EVs, and uncovers the functional tasks accomplished by the cargo of these necroptotic EVs.

## Introduction

Necroptosis is a well-studied form of regulated necrosis, defined as receptor-interacting serine/threonine-protein kinase 3 (RIPK3)-/mixed lineage kinase domain-like (MLKL)-dependent, caspase-independent cell death [1–4].

The ligation of tumor necrosis factor-α (TNF-α) results in caspase-8 cleavage and activation and, finally, apoptosis [5–7]. In these circumstances, caspase-8 cleaves and inactivates RIPK1 and RIPK3 to block necroptosis [8, 9]. However, caspase-8 suppression unleashes RIPK1 and RIPK3 inhibition. Consequently, auto- and trans-phosphorylation between RIPK1 and RIPK3 leads to the aggregation and phosphorylation of MLKL by RIPK3 [10–13]. Phosphorylated MLKL (pMLKL) then translocates to the plasma membrane to compromise membrane integrity and execute necroptosis [14]. Necroptosis is morphologically similar to necrosis, and driving the release of danger-associated molecular patterns (DAMPs), such as interleukin-33 [1, 4, 15]. Hence, necroptosis is considered an inflammatory form of cell death.

Following our [16] and others’ [17, 18] discovery that necroptotic cells, as the apoptotic cells, expose phosphatidylserine (PS) to the outer plasma membrane prior to its rupture, we found that PS-exposing necroptotic cells release extracellular vesicles (EVs) [16]. Gong et al. revealed that a member of the endosomal sorting complexes required for transport (ESCRT), ESCRTIII, plays a role in shedding of PS-exposing plasma membrane bubbles during necroptosis. ESCRTIII-mediated membrane shedding during necroptosis delayed cell death and enabled longer inflammatory signaling [17, 19]. These results underline a major knowledge gap, as the exact protein content, as well as the biogenesis and impact, of necroptotic EVs, have not yet been elucidated.

EVs are cell-derived membranous structures, divided into exosomes and microvesicles [20]. Exosomes are generated via the endosomal system as intraluminal vesicles (ILVs) that are formed during the maturation of an multivesicular endosome (MVE) and secreted by MVE fusion with the plasma membrane [21]. Microvesicles originate from the direct shedding of microdomains of the plasma membrane [22]. While originally thought to function mainly in the elimination of cellular waste, it is now understood that the fate of EVs is much more complex, mediating cell-to-cell communication in numerous settings and mechanisms [20].

Overall, this highlights the need for a large-scale proteomic analysis of necroptotic EVs to define both the upstream necroptotic mechanisms and the downstream effects of these EVs. To this end, we utilized mass spectrometry (MS)-based proteomics to characterize EVs extracted from necroptotic cells. This study sheds light on new potential players in necroptotic signaling and suggests a new mechanism mediating necroptosis-induced inflammation.

## Results

### Necroptotic PS-positive EV content shares similarity with exosome content databases

**Fig. 1:**
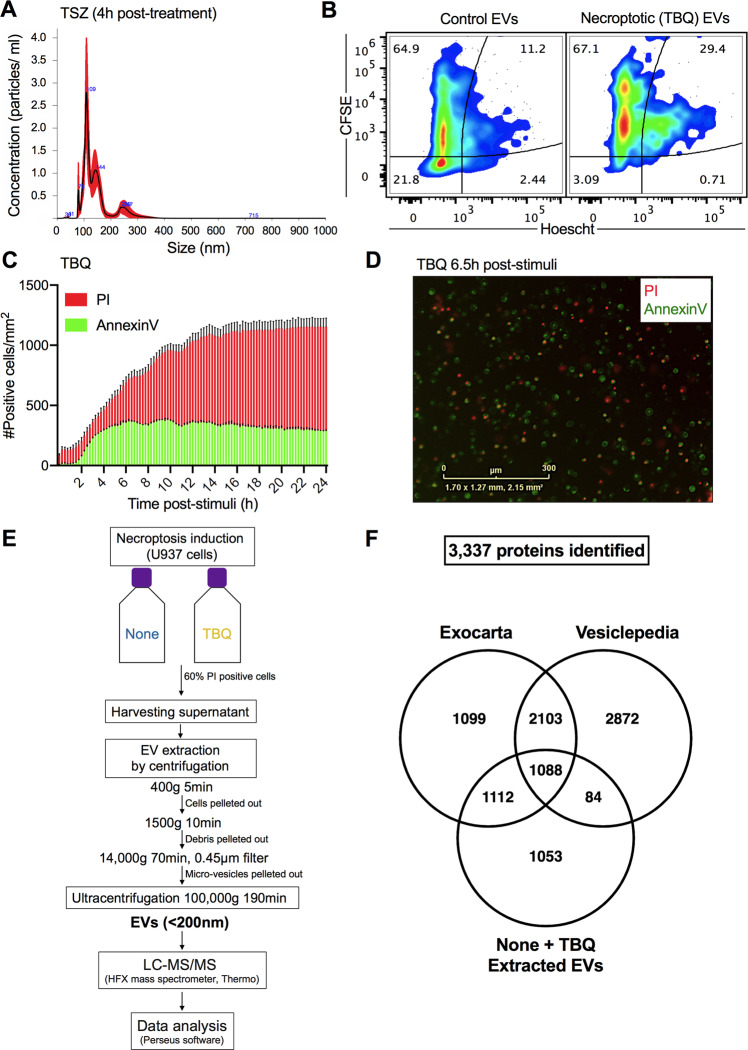
Extraction of necroptotic extracellular vesicles (EVs). **A** U937 cells were treated with TNF-α (1.15 nM), SMAC (AZD5582, 2.5 mM), and z-VAD-fmk (20 mM) (denoted TSZ) to induce necroptosis. After 4 h, EVs were extracted using ultracentrifugation and analyzed for size and concentration by NanoSight. **B** CFSE-stained U937 cells were treated with TNF-α (1.15 nM), Birinapant (5 mM), and QVD-OPh (20 mM) (denoted TBQ) to induce necroptosis. Five hours later, EVs were extracted using ultracentrifugation, stained with Hoescht33342, and analyzed by flow cytometry (Attune NxT) for CFSE and Hoescht fluorescence intensity to examine their protein and nucleic acid composition, respectively. **C**, **D** U937 cells were treated with TBQ as above to induce necroptosis. Cell viability was monitored using AnnexinV-FITC (green) and PI (red) via real-time imaging (IncucyteZoom). **C** Number of AnnexinV- and PI-positive cells per mm^2^ at the indicated time points post stimuli. **D** Representative image of treated cells at 6.5 h post stimuli. **E** Schematic overview of the experimental and data analysis procedure. U937 cells were left untreated as a control (denoted none) or stimulated for necroptosis (TBQ). When TBQ-treated cells reached 60% PI positivity, supernatants were harvested for serial centrifugation and ultracentrifugation. Six pairs of independently extracted EVs were analyzed as biological replicates by mass spectrometry and Perseus data analysis software. **F** 3337 proteins were identified in the extracted EVs from both control (none) and necroptotic (TBQ-treated) cells by mass spectrometry. Venn diagram of total proteins identified in either none or TBQ-extracted EVs compared with the exosome proteome data bases, ExoCarta and Vesiclepedia. **A**, **C**, **D** Data are representative of at least three independent experiments. **A** Data are presented as the mean of five acquired samples ±SEM and representative of three independent experiments. **B** Plots are representative of duplicate samples. **C** Data are presented as the mean of triplicate samples ±SD. EVs, extracellular vesicles; TSZ, TNF-α, SMAC, and z-VAD-fmk; TBQ, TNF-α, Birinapant, and QVD-OPh; PI, propidium iodide; h, hours; LC-MS/MS, liquid chromatography with tandem mass spectrometry; SEM, standard error of the mean; SD, standard deviation.

PS exposure on the outer cell membrane was long thought of as a phenomenon restricted to apoptosis [23]. We, and others, have challenged this dogma by demonstrating that necroptotic cells expose PS on the outer cell membrane and release PS-exposing EVs [16–18, 24, 25]. These necroptotic EVs are 191.7 ± 8 nm in size, according to nanoparticle tracking analysis (NTA) (Fig. 1A), corresponding with the size of exosomes (50–150 nm) and microvesicles (50–500 nm) [20]. In addition, protein labeling and nucleotide labeling, revealed that necroptotic EVs are rich with protein cargo (Fig. 1B). Thus, we aimed to characterize the proteome of necroptotic EVs by utilizing MS-based proteomics.

In order to correctly time the extraction of necroptotic EVs, we first calibrated necroptotic cell death kinetics in U937 cells. Cells were treated with TNF-α, Birinapant (a SMAC mimetic), and the pan-caspase inhibitor QVD-OPh (denoted TBQ) to induce necroptosis. Necroptotic cells stain with AnnexinV in a time-dependent manner before becoming propidium iodide (PI) positive, confirming that, during necroptosis, PS exposure on the outer plasma membrane precedes membrane rupture (Fig. 1C). In support of this, AnnexinV-single-positive cells were detected for 6.5 h following necroptosis induction (Fig. 1D). Based on these data, we scheduled the EV extraction for when AnnexinV positivity has plateaued and membrane rupture, i.e., PI positivity, is starting to peak (5–6.5 h post stimuli).

To investigate the protein composition of necroptotic EVs, supernatants from untreated or necroptotic cells were harvested, and a series of centrifugations were performed to pellet the cells, cell debris, and microvesicles. Finally, ultracentrifugation was conducted to pellet the EVs for consequent analysis by liquid chromatography with tandem mass spectrometry (LC-MS/MS). Data were then quantified by a label-free quantification (LFQ) approach and analyzed using Perseus software (Fig. 1E). For the complete proteome analysis, six pairs of independently extracted EVs from untreated and necroptotic cells were analyzed as biological replicates.

A total of 3337 proteins were identified in the extracted EVs from both control and necroptotic cells. To test our experimental method in light of current published data regarding EVs content, we compared the total proteins identified in both control and necroptotic EVs with the human exosome proteome databases, ExoCarta and Vesiclepedia, using Venn analysis. Out of 3337 proteins, 2200 were present in the ExoCarta database, which constitutes 66% of the total protein content of the extracted EVs. In addition, the extracted EVs shared 1172 common proteins with the Vesiclepedia database, i.e., 35% of their total protein content (Fig. 1F). This includes 65 of the 75 most frequently identified proteins in both databases (Supplementary Fig. S2 and Table S1). Among these are EV biogenesis factors, e.g., Clathrin heavy chain 1 (CLTC) and the ESCRT accessory proteins programmed cell death 6-interacting protein (PDCD6IP, also known as Alix), as well as tetraspanins, such as CD63 and Flotillin-1 (FLOT1), and intracellular EV trafficking proteins from the annexin and Ras-related protein families [20]. Notably, 1053 proteins are unique to our system.

These results support the validity of our EV extraction system, making our data comparable with established vesicle proteome research, and presenting additional unique content to be studied.

### Necroptotic EVs feature a unique proteome signature

EVs are thought to have a role in cell maintenance via the dumping of unwanted cellular content [26]. In support, interfering with necroptotic EV release by silencing the ESCRTIII family member, charged multivesicular body protein 2A (CHMP2A), or Rab27a and Rab27b that are required for exocytosis, increases the sensitivity of cells to necroptosis [17, 18]. In addition, EVs are known to function in cell-to-cell communication [20]. Therefore, we hypothesized that EV formation, cargo selection, and release during necroptosis are highly regulated and selective, and exclusively characterize the necroptotic EVs compared with the control EVs.

**Fig. 2:**
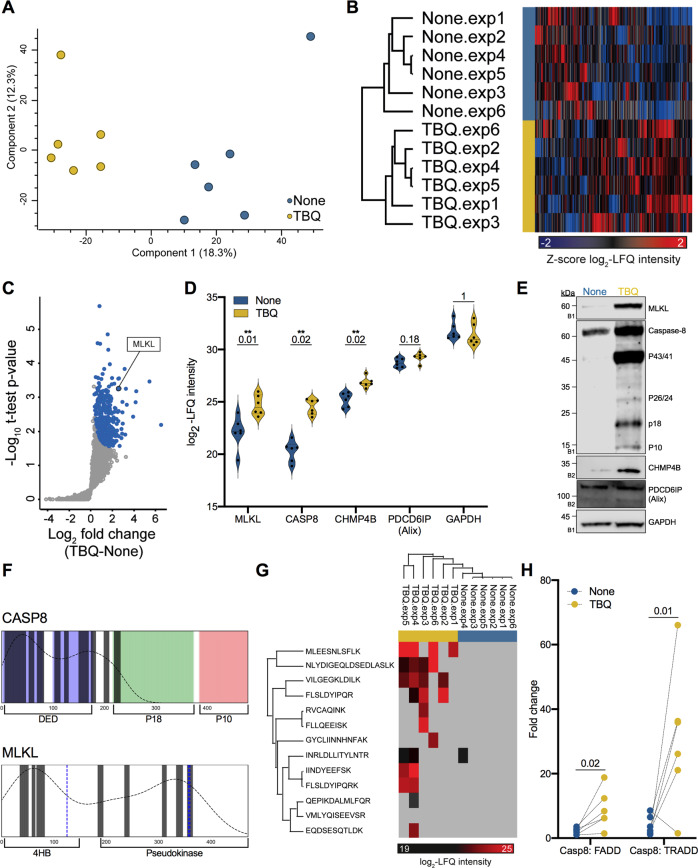
Proteome distinguishes between control and necroptotic EVs. **A** Principal component analysis of biological replicates of extracted EVs showing that samples separate based on treatment (none vs. TBQ). **B** Unsupervised hierarchical clustering analysis of 2984 identified proteins between biological replicates, named by EV extraction. Color represents the *Z*-score of log_2_-LFQ intensity of each protein. **C** Volcano plot representing a one-sided *t*-test analysis of necroptotic (TBQ) vs. control (none) EVs. 352 proteins were significantly upregulated in the necroptotic EVs and will be termed “TBQ EV-enriched proteins” (blue, FDR cutoff = 0.1, S0 = 0.1). MLKL is marked. **D** Violin plots showing log_2_-LFQ intensity of selected identified proteins. *Q*-value is mentioned individually above each plot, ^*^Q < 0.1, ^**^Q < 0.05. **E** Validation of the selected EV proteins from (**D**) by immunoblot analysis of EVs from untreated and TBQ-treated cells using antibodies against MLKL, caspase-8, CHMP4B, PDCD6IP (Alix), and GAPDH. **F** Alignment of the full-length caspase-8 and MLKL protein structures with peptides detected by MS analysis (marked as bars). Key structures of caspase-8 are colored and labeled: DED domains in blue, p18 subunit in green, and p10 subunit in red. Key structures of MLKL are labeled and phosphorylation sites are marked in blue. The dotted curve represents the density of the detected peptides along the full protein structure. **G** Hierarchical clustering analysis of caspase-8 peptides detected by MS analysis. Color represents the log_2_-LFQ intensity of each peptide, gray represents undetected levels. The few peptides that are not aligned with the DED domains, i.e., EQDSESQTLDK and GYCLIINNHNFAK, are detected only in a single sample. **H** Dot plot showing the fold change in LFQ intensity between caspase-8 and FADD, or TRADD, paired by experiment. *p*-Value for the multiple *t*-test is shown. **E** EVs for validation were extracted independently of the six pairs of biological replicates that were analyzed by mass spectrometry. The number on the left of each blot indicates the blot number, each corresponding to an independent EV extraction. EVs, extracellular vesicles; TBQ, TNF-α, Birinapant, and QVD-OPh; LFQ, label-free quantification, CASP8, caspase-8; FDR, false discovery rate; 4HB, N-terminal four-helix bundle.

To test this hypothesis, we performed principal component analysis between biological replicates. This analysis revealed that samples separate based on treatment (none vs. TBQ), supporting the biological interest in characterizing the necroptotic EV-specific cargo (Fig. 2A). Furthermore, unsupervised hierarchical clustering of 2984 identified proteins, which separated samples into two distinct groups, indicates that the necroptotic EVs are characterized by a distinct proteome signature (Fig. 2B). Statistical analysis using a one-sided Student’s *t*-test for paired samples yielded 352 proteins significantly upregulated in necroptotic vs. control EVs, with a false discovery rate (FDR) cutoff of 0.1 and S0 cutoff of 0.1, from here on termed “TBQ EV-enriched proteins” (Fig. 2C). TBQ EV-enriched proteins share 261 and 107 proteins with the exosome proteome databases, ExoCarta and Vesiclepedia, respectively, and contain 84 unique proteins (Supplementary Fig. S3). To validate these findings, we performed immunoblot analysis to some of the unique proteins: MLKL, caspase-8, and the ESCRTIII member, CHMP4B (Fig. 2D). We found that MLKL, caspase-8, and CHMP4B are indeed detected at higher levels in necroptotic EVs compared with control EVs. The levels of the exosome marker, PDCD6IP (Alix), and GAPDH, which the proteomics data showed a non-significant fold change, were unchanged (Fig. 2E).

Furthermore, the MS detected that MLKL peptides are distributed randomly along the protein, while the capsase-8 peptides are almost entirely aligned with the death effector domain (DED)_1_-DED_2_ region, including the key residues involved in the DED-mediated filament assembly between death-inducing signaling complex (DISC) components [27] (Fig. 2F). Quantitative analysis of the detected caspase-8 peptides confirmed our immunoblotting validation, both showing high levels of the DED-containing caspase-8 forms (Fig. 2G). We further assessed the presence of other DISC or TNF signaling complex components within the extracted EVs. Although the DED-containing adaptors FAS-associated death domain protein (FADD) and TNFR1-associated death domain (TRADD), which are capable of binding caspase-8 within the DISC, were detected in the extracted EVs, no significant upregulation in the necroptotic EVs was observed (Supplementary Table S2). Comparing caspase-8 to the FADD and TRADD LFQ intensity in each sample, emphasized that the ratio between these three proteins is not maintained among the control and the necroptotic EVs (Fig. 2H). This fits into the known model of TNF-α signaling (Supplementary text).

In summary, necroptotic EVs are characterized by a unique proteome signature with 352 significantly upregulated proteins, as revealed by MS and supported by validation with immunoblotting.

### Components of ESCRTIII machinery and inflammatory signaling are enriched in the necroptotic EVs

**Fig. 3:**
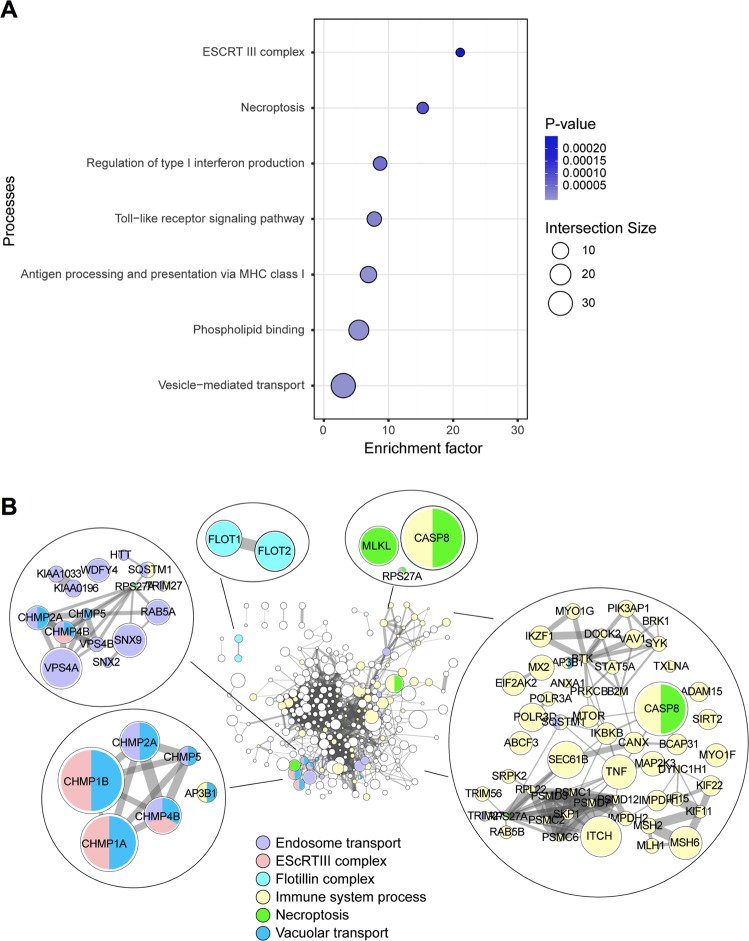
Enrichment and protein–protein interaction analyses reveal vesicle and inflammation pathways enriched in the necroptotic EVs. **A** Processes enriched in the TBQ EV-enriched proteins (Fisher’s exact test, FDR = 0.02, compared to the Vesiclepedia human proteome database). Color bars represent Fisher’s test *p*-values, symbol size represents intersection size between our dataset and the GO category. **B** Protein–protein interaction network of the TBQ EV-enriched proteins (based on the String database). Node size represents fold change of TBQ vs. control. Edge width represents protein–protein co-expression level. Marked in color are proteins belonging to selected enriched biological processes. EVs, extracellular vesicles; TBQ, TNF-α, Birinapant, and QVD-OPh; GO, gene ontology; FDR, false discovery rate.

To explore the distinctive signature of TBQ EV-enriched proteins we performed gene ontology (GO) and Kyoto encyclopedia of genes and genomes (KEGG) pathways enrichment analysis (Fig. 3A and Supplementary Table S3). We used the list of EV proteins from the Vescilepedia database as the background against which the TBQ EV-enriched proteins were tested (Fisher’s exact test, Benjamini-Hochberg FDR < 0.02). Thus, the enriched processes represent the distinct signature of the necroptotic EVs in comparison to other EV systems, not to the general human proteome. As predicted, the “necroptosis” process was significantly enriched, supporting the specificity of the experimental system.

As opposed to apoptotic cells, whose content is well-contained and rapidly cleared by phagocytosis, necroptotic cells profligately release cellular content as DAMPs following membrane permeabilization, triggering inflammation [4]. We surmised that the inflammatory potential of necroptosis may, in part, be due to the contribution of EVs. In support of this, GO biological processes that are significantly enriched in necroptotic EVs include inflammatory signaling pathways, such as the “Toll-like receptor signaling pathway” and the “regulation of type I interferon production”. The ESCRT family is a group of proteins that facilitates protein transport [28]. The ESCRTIII members, CHMP2A and CHMP4B, were found to colocalize with pMLKL near the plasma membrane during necroptosis, thus suggesting a function in the release of necroptotic EVs [17–19, 24]. In agreement, the cellular compartment annotated as “ESCRTIII” was enriched in necroptotic EVs.

Next, to get further insight into the biological events governing necroptotic EVs and their connectivity, we generated a protein–protein interaction network of the TBQ EV-enriched proteins based on the String protein–protein interaction database. We found well-documented connections between proteins that function in vesicle formation, transport, and release, as well as in inflammation (Fig. 3B). As these connections are established in current literature, this suggests that EV formation and release during necroptosis is also a regulated and selective process.

Overall, this global profiling of the protein cargo of necroptotic EVs indicates possible mechanisms for their upstream formation and release, and their downstream binding, uptake, and inflammatory effects on recipient cells.

### Necroptotic EVs contain new unstudied components of vesicle formation and transport, and necroptosis signaling pathways

**Fig. 4:**
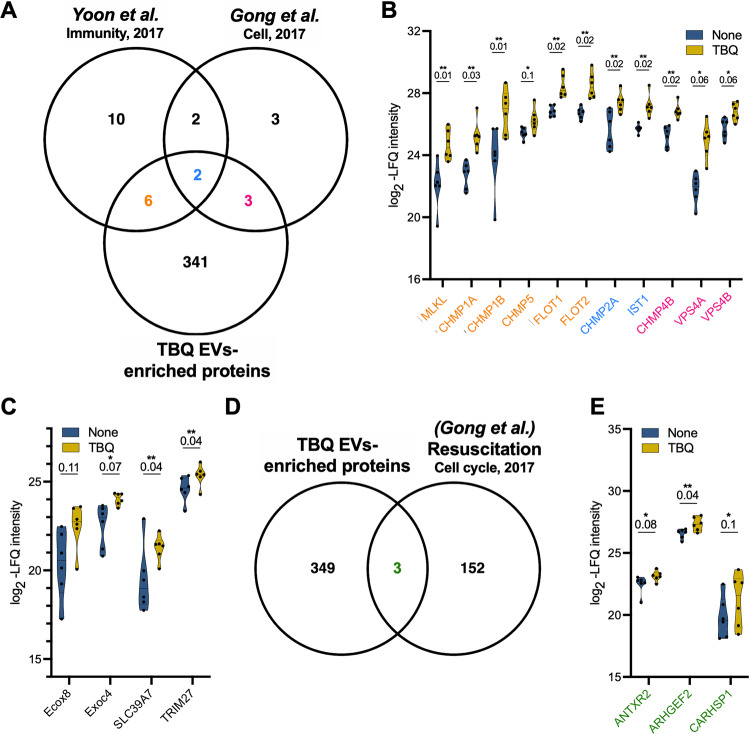
Necroptotic EVs are abundant with reported and unreported components of vesicle formation and transport and necroptosis signaling pathways. **A** Venn diagram of the TBQ EV-enriched proteins compared with proteins that were found using mass spectrometry to associate with MLKL within EVs by Yoon et al. [18] and with proteins that were shown to regulate EV formation and sensitivity to necroptosis by Gong et al. [17]. **B** Violin plots showing log_2_-LFQ intensity of overlapping proteins. Protein names are colored according to their overlapping groups in (**A**). *Q*-value is mentioned individually above each plot, ^*^Q < 0.1, ^**^Q < 0.05. **C** Violin plots showing log_2_-LFQ intensity of selected proteins that were upregulated in necroptotic (TBQ) vs. control (none) EVs. *Q*-value is mentioned individually above each plot, ^*^Q < 0.1, ^**^Q < 0.05. **D** Venn diagram of the TBQ EV-enriched proteins compared with genes that were found by RNA-Seq to be upregulated during resuscitation from necroptosis by Gong et al. [19]. **E** Violin plots showing log_2_-LFQ intensity of overlapping proteins from (**D**) (green). *Q*-value is mentioned individually above each plot, ^*^Q < 0.1, ^**^Q < 0.05. EVs, extracellular vesicles; TBQ, TNF-α, Birinapant, and QVD-OPh; FDR, false discovery rate.

As mentioned above, we [16] and others [17, 18], have previously demonstrated PS exposure during necroptosis. We reported that PS-exposing necroptotic cells release EVs early during necroptosis, which contain pMLKL [16]. Yoon et al. have shown that during necroptosis MLKL controls the formation of EVs and enhances their release [18]. Comparison of our TBQ EV-enriched proteins with the major proteins reported to precipitate with MLKL by Yoon et al. (Fig. 4A) revealed eight proteins in common, including the ESCRTIII members, CHMP1A, CHMP1B, CHMP2A, CHMP5, IST-1, and the lipid raft-associated proteins [29], flotillin-1 and flotillin-2 (Fig. 4B). Interestingly, it has recently been shown that flotillin-1 and flotillin-2 delay necroptosis by removing pMLKL from membranes [30]. Their presence within the necroptotic EVs, confirmed by both our and Yoon et al.’s systems, suggests that this mechanism might not solely culminate in MLKL degradation but also in its exocytosis. Gong et al. revealed, by silencing many ESCRT family members, that the ESCRTIII complex acts downstream of pMLKL to induce the shedding of MLKL-damaged membrane, consequently delaying cell death execution [17]. In agreement, five of ESCRT family member proteins were significantly upregulated in our necroptotic EVs, including two that were also found in the analysis by Yoon et al. (Fig. 4A, B).

Beyond the overlap with established necroptosis systems, we searched using a less stringent cutoff analysis for additional biologically relevant proteins that were upregulated in necroptotic EVs. This revealed proteins such as exocyst complex component-8 (Exoc8, also known as Exo84), Exoc4 (also known as Sec8), solute carrier family 39 member 7 (SLC39A7), and tripartite motif containing 27 (TRIM27) (Fig. 4C). Exoc8 and Exoc4 are components of the exocyst complex that tethers vesicles to the plasma membrane, resulting in their soluble M-ethylmaleimide-sensitive factor attachment protein receptor (SNARE)-mediated fusion and secretion [31] (Supplementary Table S4). Moreover, the exocyst complex binds Rab proteins on vesicles and is considered a Rab effector. Several Rab proteins were identified in the necroptotic EVs (Supplementary Table S5), and among those significantly upregulated were RAB5A, RAB5B, RAB5C, and RABGAP1. In support, Yoon et al. have shown that RNA silencing of the Rab proteins, RAB27A and RAB27B, in necroptotic cells results in a reduced number of EVs and increased cell death [18]. Thus, these mechanisms may function in necroptotic EV biogenesis.

The zinc transporter, SLC39A7, which was significantly upregulated in the necroptotic EVs, was found to be needed for TNF-α-induced necroptosis in KBM7 cells by modulating TNFR1 trafficking from the endoplasmic reticulum (ER) to the cell surface [32]. TRIM27, which we also found to be significantly upregulated in the necroptotic EVs, positively regulates TNF-α-induced apoptosis by inducing ubiquitin-specific-processing protease 7 (USP7)-mediated RIPK1 deubiquitination [33]. Thus, TRIM27 might be similarly required for necroptosis when capsase-8 is inhibited. These findings suggest that the association between necroptotic EV release and the delayed execution of cell death might be also attributed to not only MLKL shedding but also to new unstudied proteins.

Next, we compared the TBQ EV-enriched proteins with the genes that were found by Gong et al. [17] to be upregulated during necroptosis resuscitation (Fig. 4D, E). Interestingly, we found that mRNA transcripts from only three proteins were upregulated in the resuscitated necroptotic cells. One of the three proteins is calcium-regulated heat-stable protein 1 (CARHSP1), a cytoplasmic protein that localizes to exosomes to increase TNF-α production by enhancing TNF-α mRNA stability [34]. This result suggests that proteins with a key role in necroptosis are secreted in EVs, while resuscitation-supporting proteins are not. However, this finding might also be influenced by many other differences between the two systems.

Altogether, our results show that necroptotic EVs carry cargo involved in many relevant pathways, and also contain numerous proteins that have not yet been studied in this context.

### Necroptotic EVs are phagocytosed to promote inflammation in recipient cells

**Fig. 5:**
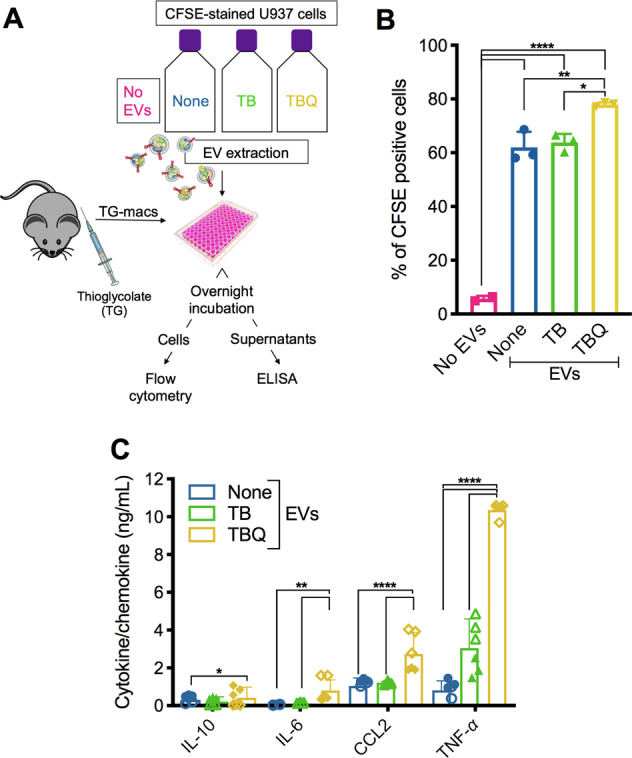
Necroptotic EVs modulate cytokine and chemokine secretion. **A** Schematic overview of EV phagocytosis assay. EVs from untreated (none), apoptotic (TB), or necroptotic (TBQ) CFSE-stained U937 cells were added to TG-macs overnight. **B** TG-macs were then harvested and analyzed by flow cytometry for their CFSE positivity, demonstrating uptake of the EVs. **C** Supernatants were collected, and secreted cytokine and chemokine levels were analyzed by ELISA. **B** Data are presented as the mean of triplicate samples ±SD. ^*^p < 0.05, ^**^p < 0.01, ^****^p < 0.0001 (one-way ANOVA followed by Tukey’s multiple-comparison test). **C** Empty symbols are triplicate samples of mouse#1, colored symbols are triplicate samples of mouse#2. Data are presented as the mean of duplicate mice ±SD, ^*^p < 0.05, ^**^p < 0.01, ^****^p < 0.0001 (two-way ANOVA followed by Tukey’s multiple-comparison test), and are representative of two independent experiment, *n* = 5. TG-macs, thioglycolate-derived peritoneal macrophages; EVs, extracellular vesicles; T, TNF-α; TB, TNF-α and Birinapant; TBQ, TNF-α, Birinapant, and QVD-OPh; PI, propidium iodide; h, hours; SD, standard deviation.

We previously reported that PS-exposing necroptotic cells are phagocytosed and trigger the secretion of TNF-α and the potent monocyte-attracting chemokine, CCL2 (MCP-1), at higher levels than following the uptake of early apoptotic cells [16]. To assess whether necroptotic EVs share this potential, we utilized our established system to examine uptake of necroptotic EVs by thioglycolate-derived peritoneal macrophages (TG-macs) and its effect (Fig. 5A). While EVs were phagocytosed by TG-macs as efficiently as control and apoptotic EVs (Fig. 5B), they induced an increased secretion of the pro-inflammatory cytokines IL-6 and TNF-α, as well as the chemokine CCL2 (Fig. 5C). These results suggest that necroptotic EVs can be taken up by recipient cells, promoting inflammatory signaling.

### Necroptotic EVs carry cancer neoantigens

**Fig. 6:**
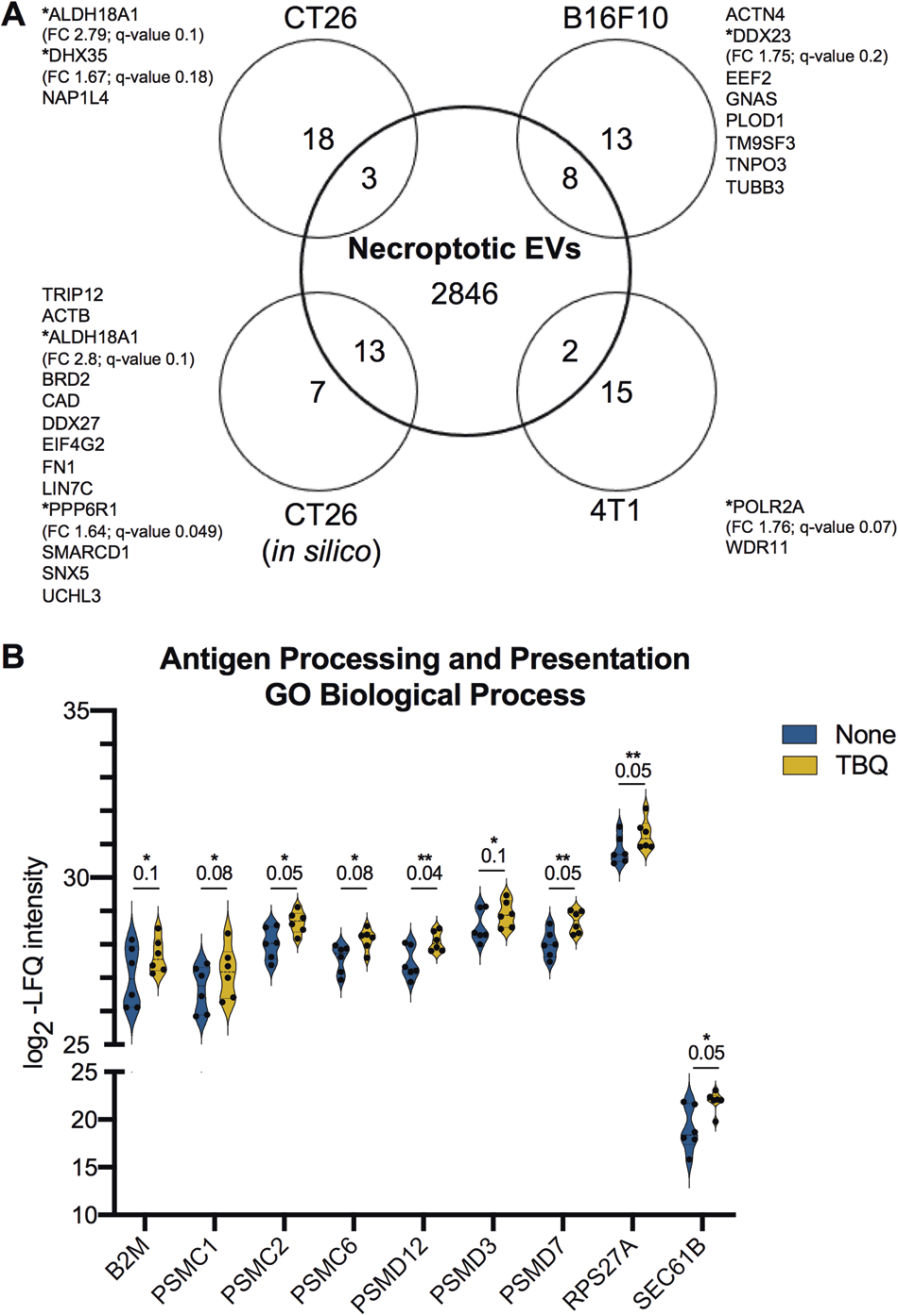
Necroptotic EVs contain tumor neoantigens. **A** Venn diagram of total proteins identified in necroptotic EVs compared with the published tumor neoantigens from B16F10, CT26, 4T1 models or CT26 in silico prediction [58]. TBQ EV-enriched proteins with FDR cutoff of 0.2 are marked with *. Fold change (FC) and *Q*-value are mentioned for each. **B** Violin plots showing log_2_-LFQ intensity of the TBQ EV-enriched proteins annotated as the enriched GO biological process “antigen processing and presentation via MHC class I”. *Q*-value is mentioned individually above each plot, ^*^Q < 0.1, ^**^Q < 0.05. EVs, extracellular vesicles; FDR, false discovery rate; FC, fold change; TBQ, TNF-α, Birinapant, and QVD-OPh; GO, gene ontology; LFQ, label-free quantification; MHC, major histocompatibility complex.

Recently, necroptosis has been suggested as a potential therapeutic mechanism to overcome the ability of tumor cells to escape apoptosis [35, 36]. In parallel, the inflammatory nature of necroptosis has been utilized to this end by few groups, who have reported that administration of necroptotic cancer cells drives anti-tumorigenic adaptive immune responses [35–39]. Nevertheless, the exact mechanism of this effect is not yet fully understood. In this regard, proteins delivered by exosomes are internalized into recipient dendritic cells, processed, and presented as antigens, potentially triggering an immune response [40]. Thus, to address the potential of necroptotic EVs to deliver tumor neoantigens, we compared all the proteins identified in the necroptotic EVs with neoantigens in a dataset derived from B16F10, CT26, 4T1, or CT26 cell lines or in an in silico prediction model, which contains immunogenic antigens recognized by CD4^+^ T cells that elicit an anti-tumor response. We found a total of 26 potential neoantigens in our necroptotic EVs, with six of them being significantly, or almost significantly, upregulated in comparison to control EVs (Fig. 6A).

Mast cell- and macrophage-derived EVs contain signals that stimulate activation of recipient cells into immunogenic antigen-presenting cells (APCs) [41]. In support, our analysis found that the GO biological process annotated “antigen processing and presentation of exogenous peptide antigen via major histocompatibility complex (MHC) class I” was significantly enriched in the TBQ EV-enriched proteins (Figs. 3A and 6B), suggesting that necroptotic EVs can facilitate activation and antigen presentation by APCs upon uptake and serve as a potential delivery system for tumor neoantigens.

## Discussion

Although necroptosis has been known for two decades as a regulated and inflammatory form of cell death, neither its regulation nor its inflammation-inducing consequences have been fully elucidated. Here, we uncover the proteome of necroptotic EVs using MS-based proteomics of ultracentrifugation-extracted necroptotic EVs. In this study, using MS-based proteomics of ultracentrifugation-extracted necroptotic EVs, we uncover the necroptotic EVs’ unique proteome signature.

We found that components of ESCRTIII machinery and inflammatory signaling are enriched in necroptotic EVs. Interestingly, we found that many ESCRTIII members are upregulated in necroptotic EVs, some of which were first shown by Gong et al. to play a role in decreasing sensitivity to necroptosis and enhancing recovery from early necroptosis [17]. This is a key finding, bridging the system of Gong et al. and our previous report of EV release during necroptosis [16].

Among the enriched processes in necroptotic EVs, we also found the molecular function group “phospholipid binding” that includes many annexin family members. Annexins bind phospholipids in a calcium-dependent manner and function in exocytosis and vesicle secretion. Annexins are also suggested to have a role in vesicle docking to recipient cells [20]. Therefore, our finding proposes the involvement of annexins in both the release of PS-exposing necroptotic EVs and their recognition by recipient cells.

Our results strongly imply that there are still unknown connections to be established as necroptotic EV research develops. We found that flotillin-1 and flotillin-2, which are lipid raft-associated proteins, are significantly upregulated in necroptotic EVs, suggesting that lipid rafts play a role in the release of necroptotic EVs. In agreement, lipid-rafts are involved in PS-exposing EV release [42]. This involvement is thought to be calcium dependent in many cases, but this is mostly unclear. Transmembrane protein 16F (TMEM16F, also called ANO6), which was detected in both necroptotic and control EVs, is a calcium-dependent phospholipid scramblase [24]. TMEM16F was recently shown to be essential for PS exposure and plasma membrane repair, via the release of EVs [43], supporting calcium efflux as the bridge between PS exposure and EV release during necroptosis. In support, our results point to the involvement of several calcium-dependent vesicle-transport machineries, evidenced by the upregulated presence of annexins, Rabs, SNAREs, exocyst complex proteins, ESCRTIII members, and lipid rafts within necroptotic EVs.

Inward budding of early endosomal membrane forms ILVs that incorporate to form MVEs. Following MVE formation and transport, the final step of exosome secretion is MVE fusion to the plasma membrane, resulting in ILV release as exosomes [20]. This step is mediated in some cells by Rabs and their effectors from the exocyst complex, tethering vesicles to plasma membrane and inducing SNARE-mediated secretion [31, 34, 44]. So far, these mechanisms have not been implicated in necroptosis but should now be studied in this context in light of the upregulation of exocyst complex members, SNAREs, and Rab proteins in necroptotic EVs.

Although necroptosis is considered to be a highly inflammatory form of cell death, it has previously been shown to attenuate TNF-α- or lipopolysaccharide (LPS)-induced inflammation, by terminating the production of multiple cytokines and chemokines [45]. However, these reports do not take into account the inflammatory impact necroptotic cells have on recipient cells in the microenvironment. We have previously shown that PS-exposing necroptotic cells are phagocytosed and trigger the secretion of TNF-α and CCL2 at higher levels than apoptotic cells [16]. Here we report, for the first time to our knowledge, that necroptotic EVs can similarly be phagocytosed by macrophages, triggering an enhanced secretion of cytokines and chemokines in comparison to apoptotic EVs. This also fits into the observation that the release of necroptotic EVs appears to mediate an ESCRTIII-dependent delay in cell death execution, as well as sustain cytokine production and secretion from necroptotic cells and support necroptosis-induced inflammation [24]. Although our EV fraction should be free from contamination, it should be taken into account that some of the results may be due to a very small traces of the original TB or TBQ stimuli of U937 cells. Thus, in the future, it will be important to compare our finding regarding necroptotic EVs with the apoptotic EV protein cargo, as was done recently [46]. Finally, as vaccination with necroptotic cancer cells was shown to facilitate anti-tumor immunogenicity in mice [35], our discovery that necroptotic EVs contain tumor neoantigens, might contribute, in part, to this phenomenon.

Of note, the use of a caspase inhibitor to induce necroptosis might interfere with meaningful proteolytic events during this process. In addition, post-translational modifications (PTM), such as phosphorylation, might be missed by standard bottom-up MS sample processing approach [47]. Thus, future research should aim to use a necroptotic stimulus that does not include pharmacological caspase inhibition, and also to apply alternative proteomic approaches, e.g., PTM proteomics or semi-tryptic digest of proteins, or different labeling for improved protein identification. In addition, future studies should investigate EV cargo other than proteins, such as mRNA and miRNAs.

**Fig. 7:**
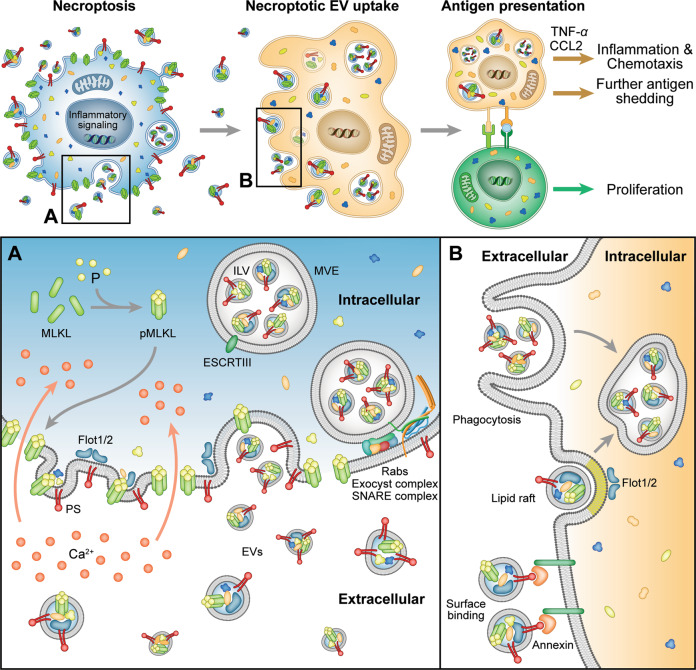
Suggested model of necroptotic EV release, uptake, and effects. **A** Necroptotic cells expose PS on the outer plasma membrane and release EVs via specific pathways, involving pMLKL-mediated Ca^2+^ influx, ESCRTIII machinery, Rabs, SNAREs, exocyst complex, and lipid rafts. **B** EVs are then taken up by recipient cells via phagocytosis, membrane fusion, or binding of PS and annexin, resulting in inflammation, chemotaxis, and antigen presentation to T cells. EVs, extracellular vesicles; PS, phosphatidylserine; pMLKL, phosphorylated MLKL; ILV, intraluminal vesicle; MVE, multivesicular endosome.

To conclude, we show that necroptotic cells release EVs via specific pathways that are yet to be fully characterized, and that these EVs can affect the microenvironment (Fig. 7). Our study establishes necroptotic EV release as a highly regulated, selective, and effective phenomenon, with the potential to shed light on cell-fate-determining players and inflammation-promoting necroptotic mechanisms. Finally, we suggest that necroptotic EVs may ultimately be harnessed for cancer vaccination.

## Materials and methods

### Cell culture

The U937 human histiocytic lymphoma cell line was cultured in RPMI-1640 (Biological industries, Beit Haemek, Israel), supplemented with 10% fetal bovine serum (FBS) (Gibco, Thermo Fisher Scientific, Waltham, MA, USA), 1% penicillin-streptomycin (Gibco) and 10 mM HEPES (Gibco), at 37 °C in a humidified 5% CO_2_ atmosphere.

### Cell death stimuli

U937 cells were seeded at 1 × 10^6^ cells/mL in FBS-free, EV-depleted, RPMI medium and were treated with TNF-α, Birinapant, and QVD-OPh (denoted TBQ) or with TNF-α, second mitochondria-derived activator of caspases (SMAC mimetic, AZD5582), and z-VAD-fmk (denoted TSZ) to induce necroptosis (Table 1), or left untreated. When indicated, cells were treated with TNF-α alone (denoted T) as a control, or with TNF-α and Birinapant (denoted TB) to induce apoptosis.

**Table 1:** Concentrations of reagents used to induce cell death.

Reagent	Concentration	Manufacturer
TNF-α	1.15 nM	PeproTech (Rocky Hill, NJ, USA)
AZD5582	2.5 μM	Tocris Bioscience (Bristol, UK)
Birinapant	2 μM	APExBIO (Houston, TX, USA)
z-VAD-fmk	20 μM	Calbiochem (Merck Millipore, Danvers, MA, USA)
QVD-OPh	20 μM	APExBIO

### Cell staining

When indicated, additional staining with propidium iodide (PI) and AnnexinV (A5) was performed (Table 2). A5 was added directly to the medium for live-cell imaging, or was added in A5 binding buffer (eBioscience, Thermo Fisher Scientific) for 10 min at room temperature before washing for flow cytometry analysis. For carboxyfluorescein succinimidyl ester (CFSE) staining, prior to addition of cell death stimuli, cells were washed twice in PBS, resuspended at 5 × 10^6^ cells/mL in PBS containing CFSE, and incubated for 10 min at 37 °C. Subsequently, five volumes of ice-cold medium were added, and cells were incubated for 5 min at 4 °C, followed by three washes, and a final resuspension in medium. Hoescht33342 was added directly to the medium.

**Table 2:** Target, dilutions, and concentrations of reagents used for staining.

Reagent	Target	Permeability	Dilution/concentration	Manufacturer
Propidium iodide (PI)	Nucleic acids	–	1 μg/mL	Sigma-Aldrich (Merck Millipore)
AnnexinV (A5)	Phosphatidylserine	–	1:200	MBL (Woburn, MA, USA)
Carboxyfluorescein succinimidyl ester (CFSE)	Cellular amines (lysine residues)	+	5 μM	Sigma‐Aldrich
Hoescht33342	Nucleic acids	+	1 μg/mL	Sigma‐Aldrich

### Cell viability assessment

To assess cell viability, 100 μL of treated and untreated cells, supplemented with PI and A5, as described above, were plated in 96-well plates in triplicate. For live-cell imaging, plates were placed on the IncucyteZOOM (Essen BioScience, Ann Arbor, MI, USA) and were recorded every 10–30 min. Data were analyzed using the IncucyteZoom2016B analysis software and exported to GraphPad Prism software.

Supplementary assessment was performed using flow cytometry. Treated and untreated cells were collected into a 96-well U-shaped plate. Cells were stained with PI and A5 as described above and re-suspended in 200 μL PBS. Samples were acquired by the flow cytometry using the Attune NxT (Thermo Fisher Scientific) and data were analyzed using FlowJo software (TreeStar, Ashland, OR, USA).

### EV extraction

In all, 2 × 10^7^ U937 cells per group were stimulated and assessed for cell death, as above. When TBQ-treated U937 cells reached 60% PI positivity by live-cell imaging and flow cytometry (4 h post TSZ-stimuli or 5–6.5 h post TBQ-stimuli), supernatants were collected and centrifuged at 400*g* for 5 min to pellet cells. Supernatants were further centrifuged at 1500*g* for 10 min to remove debris. Supernatants were then centrifuged at 14,000*g* for 70 min to remove microvesicles and filtered through a 0.45 μm filter. Finally, supernatants were centrifuged at 100,000*g* for 190 min in ultracentrifugation using type 70 Ti rotor (Oprima XL-80K, Beckman, Brea, CA, USA) to pellet the EVs (Fig. 1E). For proteomic analysis, six pairs of independently extracted EVs from untreated and TBQ-treated cells were analyzed as biological replicates by MS. For validation by immunoblot analysis, EVs were extracted in two additional independent experiments.

### NanoSight EV analysis

For a single-particle concentration and size measurements, EVs were extracted from TSZ-treated U937 cells 4 h post stimuli and analyzed using Nanoparticle Tracing Analysis (NTA) (NanoSight NS300, version 3.1.54, Malvern Instruments, Malvern, UK).

### In gel proteolysis and MS analysis

Pelleted EVs were suspended in 50 μL sodium dodecyl sulfate-polyacrylamide gel electrophoresis (SDS-PAGE) gel loading buffer (buffer composition is described below) and samples were heated at 95 °C for 5 min immediately before loading. Samples were loaded into SDS-PAGE precast gels (BIO-RAD, Rishon LeTsiyon, Israel). The proteins in the gel were reduced with 2.8 mM DTT (60 °C for 30 min), modified with 8.8 mM iodoacetamide in 100 mM ammonium bicarbonate (in the dark, at room temperature for 30 min) and digested in 10% acetonitrile and 10 mM ammonium bicarbonate with modified trypsin (Promega, Biological industries) at a 1:10 enzyme-to-substrate ratio, overnight at 37 °C. An additional second trypsinization was done for 4 h. The resulting tryptic peptides were resolved by reverse-phase chromatography on 0.075 × 200 mm fused silica capillaries (J&W) packed with Reprosil reversed-phase material (Dr Maisch GmbH, Germany). The peptides were eluted with linear 105 min gradient of 5–28% acetonitrile with 0.1% formic acid in water, 15 min gradient of 28–90% acetonitrile with 0.1% formic acid in water, and 15 min at 90% acetonitrile with 0.1% formic acid in water at flow rates of 0.15 μL/min. MS was performed by either a Q-Exactive plus or a Q-Exactive HF-X mass spectrometer (Thermo Fisher Scientific) in a positive mode using repetitively full MS scan followed by high-energy collision dissociation (HCD) of the 10 most dominant ions selected from the first MS scan.

The MS data from the biological repeats were analyzed using the MaxQuant software 1.5.2.8 version. The human part of the Uniprot database with 1% FDR in the peptide-spectrum match (PSM) and protein level [48, 49]. Protein database search was performed using the Andromeda search engine [50] and target-decoy approach [51], with an FDR cutoff of 0.01 in both the PSM and protein level.

The data were quantified by maxLFQ algorithm using the same software with the “match between runs” function enabled [52]. Briefly, peptide retention time in aligned across samples, and the inferred LFQ intensity for each protein is based on the intensity ratio of multiple samples pairs of its peptides.

Of note, Coomassie blue staining demonstrated similar stain of the control and the necroptotic EV samples, suggesting similar protein concentration among both groups (Supplementary Fig. S1A). This is supported by the fact that LFQ intensity normalization was successfully performed.

### Proteomic data analysis

Statistical analysis of the identification and quantization results was done on 3384 protein groups using Perseus software [53], filtered into 3337 unique gene names. Venn diagrams were generated using Venny 2.1.0 tool [54] (https://bioinfogp.cnb.csic.es/tools/venny/index.html) against the exosome proteome data bases, ExoCarta [55] (http://www.exocarta.org/) and Vesiclepedia [56] (http://microvesicles.org/index.html). Each dataset was selected for the human proteins, followed by filtering of duplicates.

Total proteins identified in necroptotic EVs were defined as proteins with valid quantitative values in at least three replicates of the TBQ samples.

In all analyses, values were log_2_-transformed and missing values’ imputation per sample was performed by drawing values from a normal distribution with a width of 0.5 of the sample’s valid standard deviation and a downshift of 1.4 standard deviation. LFQ intensity mentioned in text and figures are in log_2_ scale. Unless otherwise mentioned, fold change represents the raw ratio between the mean LFQ intensity values in necroptotic and control EVs.

For the unsupervised clustering analyses and principal component analysis, a minor batch effect due to sample preparation time points was removed using the R limma package [57]. For the hierarchical clustering based on protein expression, data were filtered to retain only proteins with valid quantitative values in three replicates of at least one group, resulting in 2984 proteins in total.

For the supervised analysis, one-sided Student’s *t*-test for paired samples was used with an FDR cutoff of 0.1 and S0 = 0.1 to define “TBQ EV-enriched proteins” to be used as the significantly upregulated proteins in necroptotic EVs in all downstream analyses. For enrichment analysis, a list of EV proteins was downloaded from Vescilepedia database to be used as background against which the *t*-test-significant proteins were tested (Fisher’s exact test, Benjamini-Hochberg FDR < 0.02). To identify protein–protein interactions in the TBQ EV-enriched proteins, we analyzed the list of “TBQ EV-enriched proteins” using String protein–protein interaction database (https://string-db.org/) and Cytoscape software.

### Transmission electron microscopy (TEM)

For TEM analysis, EVs from necroptotic U937 cells were extracted using a qEV Size Exclusion Column (IZon science, Medford, MA, USA) according to manufacturer’s protocol. The samples were concentrated by centrifugal filtration with 3 kDa-cutoff Amicon ultra-filtration tubes (EMD, Merck Millipore) to a final volume of 50 μL. Samples were adsorbed on Formvar/carbon coated grids and stained with 2% aqueous uranyl acetate for 30 s. Samples were examined using a JEM 1400plus TEM (Jeol, Japan).

### EV lysates for western blotting

EVs from 2 × 10^7^ treated or untreated U937 cells were extracted as above. Following ultracentrifugation, the supernatant was discarded and the pellet (containing the EVs) was lysed in 40 μL lysis buffer (150 mM NaCl, 1% (v/v) Triton X-100, 1% (w/v) sodium deoxycholate, 0.1% (w/v) SDS in 10 mM Tris-HCl, pH 7.5), supplemented with Halt protease and the phosphatase inhibitor cocktail ethylenediamine tetra-acetic acid (EDTA)-free (1:100, Pierce Biotechnology, Thermo Fisher Scientific) immediately prior to use. Following 15 min of incubation at 4 °C, lysates were centrifuged at 13,000*g* for 20 min at 4 °C. Then, 5× SDS-PAGE gel loading buffer (0.5 M DTT, 10% (w/v) SDS, 50% (v/v) glycerol, 0.2% (w/v) bromophenol blue powder, 10% (v/v) water in 1 M Tris, pH 6.8) was added to the suspension and samples were heated at 95 °C for 5 min immediately before loading.

### Western blotting

Samples were loaded into SDS-PAGE precast gels (BIO-RAD) for western blot analysis. Proteins were transferred onto 0.2 μm nitrocellulose membrane using the Trans-Blot Turbo Transfer system (BIO-RAD). Membranes were blocked with 5% skim milk in TBS-T (20 mM Tris pH 7.4, 150 mM NaCl, 0.05% Tween-20) for 1 h and probed overnight with the primary antibodies (Table 3) diluted in 5% skim milk in TBS-T. The next day, membranes were washed five times before the horseradish peroxidase (HRP)-conjugated secondary antibodies (Table 4) (diluted as above) were added for 1 h. Images were taken using Odyssey Fc system (LI-COR Biosciences, Lincoln, NE, USA) and analyzed using ImageStudio analysis software (LI-COR Biosciences).

**Table 3:** Primary antibodies used for western blots.

Primary antibody	Dilution	Source	Catalog number	Clone
Total MLKL	1:1000	Merck Millipore	MABC604	3H1, Rat IgG
Caspase-8	1:1000	R&D (Minneapolis, MN, USA)	AF1650	Polyclonal Rabbit IgG
CHMP4B	1:500	Abcam (Cambridge, UK)	Ab105767	Polyclonal Rabbit IgG
Alix (PDCD6IP)	1:1000	Cell Signaling Technology (Danvers, MA, USA)	2171	3A9, Mouse IgG1
Glyceraldehyde 3-phosphate dehydrogenase (GAPDH)	1:1000	Abcam	Ab9485	Polyclonal Rabbit IgG

**Table 4:** Secondary antibodies used for western blots.

Secondary antibody	Dilution	Source	Catalog number
HRP-conjugated goat anti-rat	1:5000	Jackson Immuno Research Laboratories (West Grove, PA, USA)	112-035-003
HRP-conjugated goat anti-rabbit	1:5000	Jackson Immuno Research Laboratories	111-035-003
HRP-conjugated donkey anti-mouse	1:5000	Jackson Immuno Research Laboratories	715-035-150

### Mice

C57BL/6J-RccHsd mice were obtained from Harlan Laboratories (Jerusalem, Israel) and grown in-house. All experiments were reviewed and approved by the Animal Care Committee of Tel Aviv University (Number 01–16–105) and were performed according to their regulations and guidelines regarding the care and use of animals for experimental procedures. Experiments were conducted in pathogen-free facilities at Tel Aviv University.

### Purification of TG-macs

Mice were injected intraperitoneally with 3 mL of 3% (v/v) thioglycolate (thioglycolic acid, Sigma-Aldrich, T3758) in PBS to elicit peritoneal macrophages using a 25 G needle. Three days later, the mice were humanely euthanized and 5 mL of PBS was injected into the peritoneal cavity of each mouse with a 21 G needle and the abdomen was gently massaged for 5–10 s. The peritoneal fluid was then collected and centrifuged at 400*g* for 5 min to pellet cells. Cells were seeded in DMEM medium, supplemented with 10% FBS (Gibco), 1% penicillin-streptomycin (Gibco), and 10 mM HEPES (Gibco) at a concentration of 1 × 10^6^ cells/mL, and then seeded at 100 μL per well in a 96-well plate the day before use. Cells were purified and seeded from five different mice, as biological replicates, in two independent experiments.

### Phagocytosis in vitro assay

For in vitro phagocytosis assays, the plate seeded with peritoneal macrophages was washed with PBS three times to discard non-adherent cells. EVs were extracted from U937 cells previously treated with either TNF-α alone (T), TNF-α and Birinapant (TB), TNF-α, Birinapant, and QVD-OPh (TBQ), or left untreated. Then, 100 μL of EVs extracted from 7.5 × 10^6^ cells were added per well of a biological triplicate of peritoneal macrophages. Following an overnight incubation, the plates were centrifuged, and supernatants were collected to measure the secretion of cytokines and chemokines by ELISA. When indicated, cells were first stained with CFSE prior to addition of cell death stimuli and EV extraction. For assessment of phagocytosis, peritoneal macrophages were harvested, following an overnight incubation, using 3 mL of 3 mM EDTA in PBS added for 10 min at room temperature, and then analyzed by flow cytometry for their CFSE positivity, demonstrating the uptake of CFSE-stained EVs.

### Enzyme-linked immunosorbent assay (ELISA)

Murine TNF-α, murine CCL2, murine IL-10, and murine IL-6 were measured using commercial ELISA kits obtained from Invitrogen, Thermo Fisher Scientific (Lower detection limits: 8, 15, 32, and 4 pg/mL, respectively), according to manufacturer’s protocol.

### Statistical analysis

Any additional statistical analysis was performed using GraphPad Prism 8 software or Python and is detailed for each experiment separately.

## Supplementary information

Supplemental Table 1

Supplemental Table 2

Supplemental Table 3

Supplemental Table 4

Supplemental Table 5

Supplemental information

Supplemental Figure 1

Supplemental Figure 2

Supplemental Figure 3

## Data Availability

The mass spectrometry proteomics data of 12 samples have been deposited to the ProteomeXchange Consortium via the PRIDE partner repository with the dataset identifier PXD018258.
