# Supplementary material for: Proteomic analysis of necroptotic extracellular vesicles
Source: Cell Death Dis. 2021 Nov 8;12(11):1059. doi: 10.1038/s41419-021-04317-z (PMC8575773; doi:10.1038/s41419-021-04317-z)
Supplement: Supplementary file 1 — Supplemental Table 1 [file 41419_2021_4317_MOESM1_ESM.pdf]

Supplementary Tables

Table S1. (Related to Fig. 1) Common exosomal marker proteins identified in the extracted EVs

Gene Name	Protein Name	Number out of 100 most identified in Exocarta	Number out of 100 most identified in Vesiclepedia
PDCD6IP	Programmed cell death 6-interacting protein	2	1
HSPA8	Heat shock cognate 71 kDa protein	3	3
GAPDH	Glyceraldehyde-3-phosphate dehydrogenase	4	2
ACTB	Actin, cytoplasmic 1	5	4
CD63	CD63 antigen	7	12
ENO1	Alpha-enolase	9	9
HSP90AA1	Heat shock protein HSP 90-alpha	10	8
TSG101	Tumor susceptibility gene 101 protein	11	39
PKM	Pyruvate kinase	12	7
LDHA	L-lactate dehydrogenase A chain	13	45
YWHAZ	14-3-3 protein zeta/delta	15	13
PGK1	Phosphoglycerate kinase 1	16	16
EEF2	Elongation factor 2	17	21
ALDOA	Fructose-bisphosphate aldolase A	18	20
HSP90AB1	Heat shock protein HSP 90-beta	19	11
ANXA5	Annexin A5;Annexin	20	10
FASN	Fatty acid synthase	21	46
YWHAE	14-3-3 protein epsilon	22	14
CLTC	Clathrin heavy chain 1	23	17
VCP	Transitional endoplasmic reticulum ATPase	26	24
TPI1	Triosephosphate isomerase	27	23
PPIA	Peptidyl-prolyl cis-trans isomerase A	28	18
MSN	Moesin	29	26
CFL1	Cofilin-1	30	25
PRDX1	Peroxiredoxin-1	31	28
PFN1	Profilin-1	32	38
RAP1B	Ras-related protein Rap-1b	33	48
ITGB1	Integrin beta-1	34	44
HSPA5	78 kDa glucose-regulated protein	35	83
SLC3A2	4F2 cell-surface antigen heavy chain	36	36
HIST1H4A	Histone H4	37	56
GNB2	Guanine nucleotide-binding protein G(I)/G(S)/G(T) subunit beta-2	38	51
ATP1A1	Sodium/potassium-transporting ATPase subunit alpha-1	39	27
YWHAQ	14-3-3 protein theta	40	40
FLOT1	Flotillin-1	41	33
FLNA	Filamin-A	42	67
CLIC1	Chloride intracellular channel protein 1	43	42
CCT2	T-complex protein 1 subunit beta	44	49
CDC42	Cell division control protein 42 homolog	45	47
YWHAG	14-3-3 protein gamma;14-3-3	46	50

	protein gamma, N-terminally processed		
GNAI2	Guanine nucleotide-binding protein G(i) subunit alpha-2	52	41
ANXA1	Annexin A11	53	43
RHOA	Transforming protein RhoA	54	74
PRDX2	Peroxiredoxin-2	56	73
GDI2	Rab GDP dissociation inhibitor beta	57	66
ACTN4	Alpha-actinin-4	59	52
YWHAB	14-3-3 protein beta/alpha;14-3-3 protein beta/alpha, N-terminally processed	60	34
RAB7A	Ras-related protein Rab-7a	61	81
LDHB	L-lactate dehydrogenase B chain;L-lactate dehydrogenase	62	35
GNAS	Guanine nucleotide-binding protein G(s) subunit alpha isoforms short	63	69
RAB5C	Ras-related protein Rab-5C	64	53
ANXA6	Annexin A6;Annexin	66	32
ANXA11	Annexin A11	67	97
KPNB1	Importin subunit beta-1	69	57
EZR	Ezrin	70	30
ACLY	ATP-citrate synthase	72	64
TFRC	Transferrin receptor protein 1	74	89
GNB1	Guanine nucleotide-binding protein G(I)/G(S)/G(T) subunit beta-1	77	37
RAN	GTP-binding nuclear protein Ran	79	72
CCT3	T-complex protein 1 subunit gamma	83	75
AHCY	Adenosylhomocysteinase	84	59
UBA1	Tubulin alpha-1A chain;Tubulin alpha-3C/D chain;Tubulin alpha-3E chain	85	68
BSG	Basigin	91	61
TCP1	T-complex protein 1 subunit alpha	95	78
MYH9	Myosin-9	99	29

1
