# Supplementary material for: Proteomic analysis of necroptotic extracellular vesicles
Source: Cell Death Dis. 2021 Nov 8;12(11):1059. doi: 10.1038/s41419-021-04317-z (PMC8575773; doi:10.1038/s41419-021-04317-z)
Supplement: Supplementary file 2 — Supplemental Table 2 [file 41419_2021_4317_MOESM2_ESM.pdf]

Table S2. (Related to Fig. 2) LFQ intensity of death-inducing signaling complex (DISC) components in the extracted EVs

Gene Name	LFQ intensity - None						LFQ intensity - TBQ						Fold change (TBQ/None)	T-test q-value
	sz168	sz170	sz171	sz186	sz187	sz189	sz168	sz170	sz171	sz186	sz187	sz189		
casp8	20.56150818	20.73325539	21.58719063	18.86445045	20.63602829	19.38393021	23.89347076	25.26086998	23.53635025	25.11215019	24.17956924	25.07266998	18.5	0.02
FADD	21.84625053	21.67556763	21.65538979	18.76533508	22.13015938	17.55722046	19.66062737	22.19426918	23.00528908	22.47507095	21.55036926	21.44597054	2.16	0.37
TRADD	21.14176178	18.02750778	18.49320412	18.63318062	18.79969025	20.5822506	18.72833061	20.07887077	22.947855	19.0666008	19.47277069	20.67425919	1.84	0.45
TRAF2	21.10859489	20.14731026	17.47186279	21.79729843	19.86410522	18.24882889	22.57074165	20.06526566	19.73435593	17.62561035	19.60129929	20.94464493	1	1
TNFRSF10B	21.67164612	19.8834877	17.70049095	18.16868973	20.30619049	20.28263092	19.10533905	19.79166985	19.50793076	20.3925705	20.7968502	20.31518936	1.24	0.66
TNFRSF1B	20.82457161	20.47545624	19.33423996	19.98062897	20.39915466	19.97661018	19.53918076	20.68142128	20.84360123	19.04958153	20.74773598	20.07275391	1.00	1.00
TNFRSF1A	Nan	Nan	Nan	Nan	Nan	Nan	Nan	Nan	Nan	Nan	Nan	Nan		
CFLAR (FLIP)	Nan	Nan	Nan	Nan	Nan	Nan	Nan	Nan	Nan	Nan	Nan	Nan		
RIPK1	Nan	Nan	Nan	Nan	Nan	Nan	Nan	Nan	Nan	Nan	Nan	Nan		
RIPK3	Nan	Nan	Nan	Nan	Nan	Nan	Nan	Nan	Nan	Nan	Nan	Nan		
