# Supplementary material for: Proteomic analysis of necroptotic extracellular vesicles
Source: Cell Death Dis. 2021 Nov 8;12(11):1059. doi: 10.1038/s41419-021-04317-z (PMC8575773; doi:10.1038/s41419-021-04317-z)
Supplement: Supplementary file 3 — Supplemental Table 3 [file 41419_2021_4317_MOESM3_ESM.pdf]

Table S3. (Related to Fig. 3) TBQ EVs-enriched proteins assigned to the enriched GO and KEGG pathways

Proteins that are also present in the Vesiclepedia database are bolded.

ESCRT III complex	Regulation of type I interferon production	Antigen processing and presentation of exogenous peptide antigen via MHC class I	Phospholipid binding	Vesicle-mediated transport	
**CHMP1A**	**DDX3X;DDX3Y**	**B2M**	**ANXA1**	**ANXA11**	KIAA1033
**CHMP1B**	**FLOT1**	PSMC1	**ANXA11**	AP3B1	LMAN1
**CHMP4B**	IKBKB	**PSMC2**	**ANXA4**	**ARF4**	LRSAM1
	ITCH	**PSMC6**	**ANXA6**	BCAP31	MYO1F
	POLR3A	**PSMD12**	**ANXA6**	**CANX**	MYO1G
	POLR3D	**PSMD3**	**ANXA7**	CCDC22	NSF
	**RPS27A;UBB;UBC**	**PSMD7**	ARHGAP9	**CHMP1A**	PACSIN3
	TRIM56	**RPS27A;UBB;UBC**	**BTK**	**CHMP2A**	PLIN3
		SEC61B	**CHMP2A**	**CHMP4B**	PPT1
			**CPNE1**	**CHMP5**	RAB5A
			**CPNE3**	COG2	RDH11
			**ESYT1**	**COPA**	RPS27A;UBB;UBC
			ESYT2	**COPB1**	RTN3
			**IQGAP2**	**CPNE1**	SNX2
			**MITD1**	**CPNE3**	SNX9
			**MYO1G**	DENND3	SQSTM1
			**PACSIN3**	**DNAJC5**	TRIM27
			SNX2	**DOCK2**	TXLNA
			**SNX9**	ESYT2	VPS11
			**WDFY4**	GOLGA3	VPS4A
				HTT	VPS4B
				KIAA0196	WDFY4

Necroptosis	Toll-like receptor signaling pathway
CASP8	**BTK**
MLKL	CASP8
**RPS27A;UBB;UBC**	**CDC2;CDK1**
	IKBKB
	**MAP2K3**
	PIK3AP1
	**RPS27A;UBB;UBC**
