# Supplementary material for: Proteomic analysis of necroptotic extracellular vesicles
Source: Cell Death Dis. 2021 Nov 8;12(11):1059. doi: 10.1038/s41419-021-04317-z (PMC8575773; doi:10.1038/s41419-021-04317-z)
Supplement: Supplementary file 4 — Supplemental Table 4 [file 41419_2021_4317_MOESM4_ESM.pdf]

**Table S4. (Related to Fig. 4) SNARE proteins identified in the necroptotic EVs**

| Gene names | Protein names | T-test q-value (FDR) | Fold change |
|---|---|---|---|
| SNAP23 | Synaptosomal-associated protein | 0.330202765 | 1.470759941 |
| VTI1B | Vesicle transport through interaction with t-SNAREs homolog 1B | 0.39383004 | 1.436236576 |
| STX3 | Syntaxin-3 | 0.438452555 | 1.414839425 |
| VAMP8 | Vesicle-associated membrane protein 8 | 0.215236025 | 1.353523461 |
| STX6 | Syntaxin-6 | 0.661335883 | 1.171636855 |
| STX7 | Syntaxin-7 | 0.730685217 | 1.142753644 |
| SEC22B | Vesicle-trafficking protein SEC22b | 0.576697035 | 1.092087572 |
| STX4 | Syntaxin-4 | 0.81756682 | 1.013655993 |
| VAMP3 | Vesicle-associated membrane protein 3 | 1 | 0.874953732 |
| VAMP7 | Vesicle-associated membrane protein 7 | 1 | 0.67976512 |
| STX8 | Syntaxin-8 | 1 | 0.453135699 |
