# Supplementary material for: Proteomic analysis of necroptotic extracellular vesicles
Source: Cell Death Dis. 2021 Nov 8;12(11):1059. doi: 10.1038/s41419-021-04317-z (PMC8575773; doi:10.1038/s41419-021-04317-z)
Supplement: Supplementary file 5 — Supplemental Table 5 [file 41419_2021_4317_MOESM5_ESM.pdf]

Table S5. (Related to Fig. 4) Rab proteins identified in increased numbers in the necroptotic EVs

Gene names	Protein names	T-test q-value (FDR)	Fold change
RAB5A	Ras-related protein Rab-5A	0.087483871	3.70098328
RAB3GAP1	Rab3 GTPase-activating protein catalytic subunit	0.109455471	2.933232655
RAB3D	Ras-related protein Rab-3D	0.197904132	2.445092994
RABGAP1;RABGAP1L	Rab GTPase-activating protein 1;Rab GTPase-activating protein 1-like, isoform 10	0.074028369	2.277118621
RAB3GAP2	Rab3 GTPase-activating protein non-catalytic subunit	0.152137097	2.276411761
RAB27A	Ras-related protein Rab-27A	0.298240786	2.253905732
RAB6A;RAB6B	Ras-related protein Rab-6A;Ras-related protein Rab-6B	0.334421893	2.02552326
RAB39A	Ras-related protein Rab-39A	0.487346251	2.020045913
RAB4A	Ras-related protein Rab-4A	0.301693431	1.750816521
RABGAP1L	Rab GTPase-activating protein 1-like	0.491986656	1.70643968
RAB5C	Ras-related protein Rab-5C	0.074357143	1.702516058
RABGEF1	Rab5 GDP/GTP exchange factor	0.388052209	1.70198044
RAB20	Ras-related protein Rab-20	0.294641884	1.573590383
RALA	Ras-related protein Ral-A	0.280316883	1.396089652
RAB11FIP1	Rab11 family-interacting protein 1	0.421975309	1.292211209
RAB18	Ras-related protein Rab-18	0.663071879	1.264642718
RAB21	Ras-related protein Rab-21	0.482321155	1.253386823
RAB27B	Ras-related protein Rab-27B	0.544103976	1.227709852
RAB11B	Ras-related protein Rab-11B	0.54047619	1.220117196
RABEP1	Rab GTPase-binding effector protein 1	0.533253685	1.208783136
RAB1B;RAB1C	Ras-related protein Rab-1B;Putative Ras-related protein Rab-1C	0.555641566	1.175131154
RAB9A	Ras-related protein Rab-9A	0.615936813	1.126404214
RAB14	Ras-related protein Rab-14	0.578848092	1.096142107
