# Supplementary material for: Proteomic analysis of necroptotic extracellular vesicles
Source: Cell Death Dis. 2021 Nov 8;12(11):1059. doi: 10.1038/s41419-021-04317-z (PMC8575773; doi:10.1038/s41419-021-04317-z)
Supplement: Supplementary file 6 — Supplemental information [file 41419_2021_4317_MOESM6_ESM.docx]

### **Supplemental information**

# **Proteomic analysis of necroptotic extracellular vesicles**

Inbar Shlomovitz^1*^, Ziv Erlich^1*^, Gali Arad^2^, Liat Edry-Botzer^1^, Sefi Zargarian^1^, Hadar Cohen^1^, Tal Manko^3^, Yifat Ofir-Birin^4^, Tomer Cooks^3^, Neta Regev-Rudzki^4^ and Motti Gerlic^1^

1. Department of Clinical Microbiology and Immunology, Sackler School of Medicine, Tel Aviv University, Tel Aviv, Israel
2. Department of Human Molecular Genetics and Biochemistry, Sackler School of Medicine, Tel Aviv University, Tel Aviv, Israel
3. The Shraga Segal Department of Microbiology, Immunology and Genetics, Ben-Gurion University of the Negev, Beer-Sheva, Israel
4. Department of Biomolecular Sciences, Weizmann Institute of Science, Rehovot, Israel

* These authors contributed equally to this work.

### **Corresponding author**

Motti Gerlic,

Department of Clinical Microbiology and Immunology, Sackler School of Medicine, Tel Aviv University, Tel Aviv 69978, Israel

Tel: +972-36409069,

E-mail: [mgerlic@tauex.tau.ac.il](mailto:mgerlic@tauex.tau.ac.il)

Supplementary text (Related to Fig. 2)

Intrigued by the presence of caspase-8 in the necroptotic EVs, we examined the specific peptides that were detected. Caspase-8 is an initiator caspase that is recruited to intracellular multi-protein complexes, such as the DISC or TNF signaling Complex I and II, following death receptor ligation(1,2). This assembly is mediated by binding of DED domains shared by caspase-8, FADD, and TRADD, resulting in homo-oligomerization, cleavage, and activation of caspase-8 and subsequent apoptosis, or necroptosis when capsase-8 activity is inhibited(3). Kinetics studies of procaspase-8 cleavage at the DISC suggest that the first cleavage event is at position D384, resulting in the formation of the p10 and p43/41 fragments, the latter of which is subsequently cleaved at position S216 to generate the p18 and p26/24 subunits(4). Treatment with QVD-OPh was shown to only partially block the first cleavage event, but to fully prevent the second cleavage event(5). In agreement, we detected mainly full-length and p43/41 caspase-8 products by immunoblot and peptides that are almost exclusively aligned with the DED domain by MS. This suggests that the p10 product remains within the necroptotic cells, while the full-length and p43/41 products are selectively secreted in necroptotic EVs. Of note, it is also possible that the second cleavage event does occur to some extent (as we detect low levels of p18 by immunoblotting), but that the remaining p26/24 is not detectable due to the specificity of the caspase-8 antibody and the detection limit of MS analysis. Following DISC formation, both the p43/41 and p26/24 subunits, which contain the DED domains, remain bound to, and contained within, the DISC(6). This corresponds with the fact that the DED-containing adaptors, FADD and TRADD, were also found in the necroptotic EVs. The fact that caspase-8 was significantly upregulated in the necroptotic EVs, while FADD and TRADD were not, matches the known stoichiometric ratios between caspase-8 and these adaptors within the DISC, as one adaptor molecule recruits many caspase-8 molecules to homo-oligomerize(4). In addition, the presence of the lipid raft-associated proteins, flotillin-1 and flotillin-2, supports the release of these membrane-bound DISC components. Notably, FLIP, RIPK1, and RIPK3 were not detected, suggesting that TNF signaling Complex II is absent from the necroptotic EVs, but this result might also stem from MS detection limits. In addition, the absence of the death receptors might be due to the ectodomain shedding reported to be involved in necroptosis(7).

## **References**

1. Fu TM, Li Y, Lu A, Li Z, Vajjhala PR, Cruz AC, et al. Cryo-EM Structure of Caspase-8 Tandem DED Filament Reveals Assembly and Regulation Mechanisms of the Death-Inducing Signaling Complex. Mol Cell [Internet]. 2016;64(2):236–50. Available from: http://dx.doi.org/10.1016/j.molcel.2016.09.009

2. Dickens LS, Boyd RS, Jukes-Jones R, Hughes MA, Robinson GL, Fairall L, et al. A Death Effector Domain Chain DISC Model Reveals a Crucial Role for Caspase-8 Chain Assembly in Mediating Apoptotic Cell Death. Mol Cell. 2012;47(2):291–305.

3. Silke J, Rickard JA, Gerlic M. The diverse role of RIP kinases in necroptosis and inflammation. Nat Immunol [Internet]. 2015 Jun 18 [cited 2016 Jan 9];16(7):689–97. Available from: http://www.ncbi.nlm.nih.gov/pubmed/26086143

4. Schleich K, Buchbinder JH, Pietkiewicz S, Kähne T, Warnken U, Öztürk S, et al. Molecular architecture of the DED chains at the DISC: Regulation of procaspase-8 activation by short DED proteins c-FLIP and procaspase-8 prodomain. Cell Death Differ. 2016;23(4):681–94.

5. Henry CM, Martin SJ. Caspase-8 Acts in a Non-enzymatic Role as a Scaffold for Assembly of a Pro-inflammatory “FADDosome” Complex upon TRAIL Stimulation. Mol Cell [Internet]. 2017;65(4):715-729.e5. Available from: http://dx.doi.org/10.1016/j.molcel.2017.01.022

6. Hoffmann JC, Pappa A, Krammer PH, Lavrik IN. A New C-Terminal Cleavage Product of Procaspase-8, p30, Defines an Alternative Pathway of Procaspase-8 Activation. Mol Cell Biol. 2009;29(16):4431–40.

7. Tanzer MC, Frauenstein A, Stafford CA, Phulphagar K, Mann M, Meissner F. Quantitative and Dynamic Catalogs of Proteins Released during Apoptotic and Necroptotic Cell Death. Cell Rep [Internet]. 2020;30(4):1260-1270.e5. Available from: https://doi.org/10.1016/j.celrep.2019.12.079

8. Zargarian S, Shlomovitz I, Erlich Z, Hourizadeh A, Ofir-Birin Y, Croker BA, et al. Phosphatidylserine externalization, “necroptotic bodies” release, and phagocytosis during necroptosis. PLoS Biol. 2017;15(6):1–23.

9. Lötvall J, Hill AF, Hochberg F, Buzás EI, Vizio D Di, Gardiner C, et al. Minimal experimental requirements for definition of extracellular vesicles and their functions: A position statement from the International Society for Extracellular Vesicles. J Extracell Vesicles. 2014;3(1):1–6.

10. Van Deun J, Mestdagh P, Agostinis P, Akay Ö, Anand S, Anckaert J, et al. EV-TRACK: Transparent reporting and centralizing knowledge in extracellular vesicle research. Nat Methods. 2017;14(3):228–32.

**Supplementary Tables**

**Table S1. (Related to Fig. 1) Common exosomal marker proteins identified in the extracted EVs**

| **Gene Name** | **Protein Name** | **Number out of 100 most identified in**  **Exocarta** | **Number out of 100 most identified in Vesiclepedia** |
| --- | --- | --- | --- |
| PDCD6IP | Programmed cell death 6-interacting protein | 2 | 1 |
| HSPA8 | Heat shock cognate 71 kDa protein | 3 | 3 |
| GAPDH | Glyceraldehyde-3-phosphate dehydrogenase | 4 | 2 |
| ACTB | Actin, cytoplasmic 1 | 5 | 4 |
| CD63 | CD63 antigen | 7 | 12 |
| ENO1 | Alpha-enolase | 9 | 9 |
| HSP90AA1 | Heat shock protein HSP 90-alpha | 10 | 8 |
| TSG101 | Tumor susceptibility gene 101 protein | 11 | 39 |
| PKM | Pyruvate kinase | 12 | 7 |
| LDHA | L-lactate dehydrogenase A chain | 13 | 45 |
| YWHAZ | 14-3-3 protein zeta/delta | 15 | 13 |
| PGK1 | Phosphoglycerate kinase 1 | 16 | 16 |
| EEF2 | Elongation factor 2 | 17 | 21 |
| ALDOA | Fructose-bisphosphate aldolase A | 18 | 20 |
| HSP90AB1 | Heat shock protein HSP 90-beta | 19 | 11 |
| ANXA5 | Annexin A5;Annexin | 20 | 10 |
| FASN | Fatty acid synthase | 21 | 46 |
| YWHAE | 14-3-3 protein epsilon | 22 | 14 |
| CLTC | Clathrin heavy chain 1 | 23 | 17 |
| VCP | Transitional endoplasmic reticulum ATPase | 26 | 24 |
| TPI1 | Triosephosphate isomerase | 27 | 23 |
| PPIA | Peptidyl-prolyl cis-trans isomerase A | 28 | 18 |
| MSN | Moesin | 29 | 26 |
| CFL1 | Cofilin-1 | 30 | 25 |
| PRDX1 | Peroxiredoxin-1 | 31 | 28 |
| PFN1 | Profilin-1 | 32 | 38 |
| RAP1B | Ras-related protein Rap-1b | 33 | 48 |
| ITGB1 | Integrin beta-1 | 34 | 44 |
| HSPA5 | 78 kDa glucose-regulated protein | 35 | 83 |
| SLC3A2 | 4F2 cell-surface antigen heavy chain | 36 | 36 |
| HIST1H4A | Histone H4 | 37 | 56 |
| GNB2 | Guanine nucleotide-binding protein G(I)/G(S)/G(T) subunit beta-2 | 38 | 51 |
| ATP1A1 | Sodium/potassium-transporting ATPase subunit alpha-1 | 39 | 27 |
| YWHAQ | 14-3-3 protein theta | 40 | 40 |
| FLOT1 | Flotillin-1 | 41 | 33 |
| FLNA | Filamin-A | 42 | 67 |
| CLIC1 | Chloride intracellular channel protein 1 | 43 | 42 |
| CCT2 | T-complex protein 1 subunit beta | 44 | 49 |
| CDC42 | Cell division control protein 42 homolog | 45 | 47 |
| YWHAG | 14-3-3 protein gamma;14-3-3 protein gamma, N-terminally processed | 46 | 50 |
| GNAI2 | Guanine nucleotide-binding protein G(i) subunit alpha-2 | 52 | 41 |
| ANXA1 | Annexin A11 | 53 | 43 |
| RHOA | Transforming protein RhoA | 54 | 74 |
| PRDX2 | Peroxiredoxin-2 | 56 | 73 |
| GDI2 | Rab GDP dissociation inhibitor beta | 57 | 66 |
| ACTN4 | Alpha-actinin-4 | 59 | 52 |
| YWHAB | 14-3-3 protein beta/alpha;14-3-3 protein beta/alpha, N-terminally processed | 60 | 34 |
| RAB7A | Ras-related protein Rab-7a | 61 | 81 |
| LDHB | L-lactate dehydrogenase B chain;L-lactate dehydrogenase | 62 | 35 |
| GNAS | Guanine nucleotide-binding protein G(s) subunit alpha isoforms short | 63 | 69 |
| RAB5C | Ras-related protein Rab-5C | 64 | 53 |
| ANXA6 | Annexin A6;Annexin | 66 | 32 |
| ANXA11 | Annexin A11 | 67 | 97 |
| KPNB1 | Importin subunit beta-1 | 69 | 57 |
| EZR | Ezrin | 70 | 30 |
| ACLY | ATP-citrate synthase | 72 | 64 |
| TFRC | Transferrin receptor protein 1 | 74 | 89 |
| GNB1 | Guanine nucleotide-binding protein G(I)/G(S)/G(T) subunit beta-1 | 77 | 37 |
| RAN | GTP-binding nuclear protein Ran | 79 | 72 |
| CCT3 | T-complex protein 1 subunit gamma | 83 | 75 |
| AHCY | Adenosylhomocysteinase | 84 | 59 |
| UBA1 | Tubulin alpha-1A chain;Tubulin alpha-3C/D chain;Tubulin alpha-3E chain | 85 | 68 |
| BSG | Basigin | 91 | 61 |
| TCP1 | T-complex protein 1 subunit alpha | 95 | 78 |
| MYH9 | Myosin-9 | 99 | 29 |

| **Gene Name** | **LFQ intensity - None** | | | | | | **LFQ intensity - TBQ** | | | | | | **Fold change (TBQ/None)** | **T-test q-value** |
| --- | --- | --- | --- | --- | --- | --- | --- | --- | --- | --- | --- | --- | --- | --- |
|  | **sz168** | **sz170** | **sz171** | **sz186** | **sz187** | **sz189** | **sz168** | **sz170** | **sz171** | **sz186** | **sz187** | **sz189** |  |  |
| casp8 | 20.56150818 | 20.73325539 | 21.58719063 | 18.86445045 | 20.63602829 | 19.38393021 | 23.89347076 | 25.26086998 | 23.53635025 | 25.11215019 | 24.17956924 | 25.07266998 | 18.5 | 0.02 |
| FADD | 21.84625053 | 21.67556763 | 21.65538979 | 18.76533508 | 22.13015938 | 17.55722046 | 19.66062737 | 22.19426918 | 23.00528908 | 22.47507095 | 21.55036926 | 21.44597054 | 2.16 | 0.37 |
| TRADD | 21.14176178 | 18.02750778 | 18.49320412 | 18.63318062 | 18.79969025 | 20.5822506 | 18.72833061 | 20.07887077 | 22.947855 | 19.0666008 | 19.47277069 | 20.67425919 | 1.84 | 0.45 |
| TRAF2 | 21.10859489 | 20.14731026 | 17.47186279 | 21.79729843 | 19.86410522 | 18.24882889 | 22.57074165 | 20.06526566 | 19.73435593 | 17.62561035 | 19.60129929 | 20.94464493 | 1 | 1 |
| TNFRSF10B | 21.67164612 | 19.8834877 | 17.70049095 | 18.16868973 | 20.30619049 | 20.28263092 | 19.10533905 | 19.79166985 | 19.50793076 | 20.3925705 | 20.7968502 | 20.31518936 | 1.24 | 0.66 |
| TNFRSF1B | 20.82457161 | 20.47545624 | 19.33423996 | 19.98062897 | 20.39915466 | 19.97661018 | 19.53918076 | 20.68142128 | 20.84360123 | 19.04958153 | 20.74773598 | 20.07275391 | 1.00 | 1.00 |
| TNFRSF1A | Nan | Nan | Nan | Nan | Nan | Nan | Nan | Nan | Nan | Nan | Nan | Nan |  |  |
| CFLAR (FLIP) | Nan | Nan | Nan | Nan | Nan | Nan | Nan | Nan | Nan | Nan | Nan | Nan |  |  |
| RIPK1 | Nan | Nan | Nan | Nan | Nan | Nan | Nan | Nan | Nan | Nan | Nan | Nan |  |  |
| RIPK3 | Nan | Nan | Nan | Nan | Nan | Nan | Nan | Nan | Nan | Nan | Nan | Nan |  |  |

Table S2. (Related to Fig. 2) LFQ intensity of death-inducing signaling complex (DISC) components in the extracted EVs

Table S3. (Related to Fig. 3) TBQ EVs-enriched proteins assigned to the enriched GO and KEGG pathways

Proteins that are also present in the Vesiclepedia database are bolded.

| **ESCRT III complex** | **Regulation of type I interferon production** | **Antigen processing and presentation of exogenous peptide antigen via MHC class I** | **Phospholipid binding** | **Vesicle-mediated transport** | |
| --- | --- | --- | --- | --- | --- |
| **CHMP1A** | **DDX3X;DDX3Y** | **B2M** | **ANXA1** | **ANXA11** | KIAA1033 |
| **CHMP1B** | **FLOT1** | PSMC1 | **ANXA11** | AP3B1 | LMAN1 |
| **CHMP4B** | IKBKB | **PSMC2** | **ANXA4** | **ARF4** | LRSAM1 |
|  | ITCH | **PSMC6** | **ANXA6** | BCAP31 | MYO1F |
|  | POLR3A | **PSMD12** | **ANXA6** | **CANX** | MYO1G |
|  | POLR3D | **PSMD3** | **ANXA7** | CCDC22 | NSF |
|  | **RPS27A;UBB;UBC** | **PSMD7** | ARHGAP9 | **CHMP1A** | PACSIN3 |
|  | TRIM56 | **RPS27A;UBB;UBC** | **BTK** | **CHMP2A** | PLIN3 |
|  |  | SEC61B | **CHMP2A** | **CHMP4B** | PPT1 |
|  |  |  | **CPNE1** | **CHMP5** | RAB5A |
| **Necroptosis** | **Toll-like receptor signaling pathway** |  | **CPNE3** | COG2 | RDH11 |
| CASP8 | **BTK** |  | **ESYT1** | **COPA** | RPS27A;UBB;UBC |
| MLKL | CASP8 |  | ESYT2 | **COPB1** | RTN3 |
| **RPS27A;UBB;UBC** | **CDC2;CDK1** |  | **IQGAP2** | **CPNE1** | SNX2 |
|  | IKBKB |  | **MITD1** | **CPNE3** | SNX9 |
|  | **MAP2K3** |  | **MYO1G** | DENND3 | SQSTM1 |
|  | PIK3AP1 |  | **PACSIN3** | **DNAJC5** | TRIM27 |
|  | **RPS27A;UBB;UBC** |  | SNX2 | **DOCK2** | TXLNA |
|  |  |  | **SNX9** | ESYT2 | VPS11 |
|  |  |  | **WDFY4** | GOLGA3 | VPS4A |
|  |  |  |  | HTT | VPS4B |
|  |  |  |  | KIAA0196 | WDFY4 |

**Table S4. (Related to Fig. 4) SNARE proteins identified in the necroptotic EVs**

| **Gene names** | **Protein names** | **T-test q-value (FDR)** | **Fold change** |
| --- | --- | --- | --- |
| SNAP23 | Synaptosomal-associated protein | 0.330202765 | 1.470759941 |
| VTI1B | Vesicle transport through interaction with t-SNAREs homolog 1B | 0.39383004 | 1.436236576 |
| STX3 | Syntaxin-3 | 0.438452555 | 1.414839425 |
| VAMP8 | Vesicle-associated membrane protein 8 | 0.215236025 | 1.353523461 |
| STX6 | Syntaxin-6 | 0.661335883 | 1.171636855 |
| STX7 | Syntaxin-7 | 0.730685217 | 1.142753644 |
| SEC22B | Vesicle-trafficking protein SEC22b | 0.576697035 | 1.092087572 |
| STX4 | Syntaxin-4 | 0.81756682 | 1.013655993 |
| VAMP3 | Vesicle-associated membrane protein 3 | 1 | 0.874953732 |
| VAMP7 | Vesicle-associated membrane protein 7 | 1 | 0.67976512 |
| STX8 | Syntaxin-8 | 1 | 0.453135699 |

**Table S5. (Related to Fig. 4) Rab proteins identified in increased numbers in the necroptotic EVs**

| **Gene names** | **Protein names** | **T-test q-value (FDR)** | **Fold change** |
| --- | --- | --- | --- |
| RAB5A | Ras-related protein Rab-5A | 0.087483871 | 3.70098328 |
| RAB3GAP1 | Rab3 GTPase-activating protein catalytic subunit | 0.109455471 | 2.933232655 |
| RAB3D | Ras-related protein Rab-3D | 0.197904132 | 2.445092994 |
| RABGAP1;RABGAP1L | Rab GTPase-activating protein 1;Rab GTPase-activating protein 1-like, isoform 10 | 0.074028369 | 2.277118621 |
| RAB3GAP2 | Rab3 GTPase-activating protein non-catalytic subunit | 0.152137097 | 2.276411761 |
| RAB27A | Ras-related protein Rab-27A | 0.298240786 | 2.253905732 |
| RAB6A;RAB6B | Ras-related protein Rab-6A;Ras-related protein Rab-6B | 0.334421893 | 2.02552326 |
| RAB39A | Ras-related protein Rab-39A | 0.487346251 | 2.020045913 |
| RAB4A | Ras-related protein Rab-4A | 0.301693431 | 1.750816521 |
| RABGAP1L | Rab GTPase-activating protein 1-like | 0.491986656 | 1.70643968 |
| RAB5C | Ras-related protein Rab-5C | 0.074357143 | 1.702516058 |
| RABGEF1 | Rab5 GDP/GTP exchange factor | 0.388052209 | 1.70198044 |
| RAB20 | Ras-related protein Rab-20 | 0.294641884 | 1.573590383 |
| RALA | Ras-related protein Ral-A | 0.280316883 | 1.396089652 |
| RAB11FIP1 | Rab11 family-interacting protein 1 | 0.421975309 | 1.292211209 |
| RAB18 | Ras-related protein Rab-18 | 0.663071879 | 1.264642718 |
| RAB21 | Ras-related protein Rab-21 | 0.482321155 | 1.253386823 |
| RAB27B | Ras-related protein Rab-27B | 0.544103976 | 1.227709852 |
| RAB11B | Ras-related protein Rab-11B | 0.54047619 | 1.220117196 |
| RABEP1 | Rab GTPase-binding effector protein 1 | 0.533253685 | 1.208783136 |
| RAB1B;RAB1C | Ras-related protein Rab-1B;Putative Ras-related protein Rab-1C | 0.555641566 | 1.175131154 |
| RAB9A | Ras-related protein Rab-9A | 0.615936813 | 1.126404214 |
| RAB14 | Ras-related protein Rab-14 | 0.578848092 | 1.096142107 |

**Supplemental Figure legends**

Figure S1. (Related to materials and methods section “In gel proteolysis and mass spectrometry analysis”) Coomassie blue staining and TEM of the necroptotic EVs

**A**, Coomassie blue staining of the control and the necroptotic EVs. **B**, Necroptotic EVs extracted by qEV Size Exclusion Column (IZon science) were prepared for transmission electron microscope (TEM) and images were captured on the JEM 1400plus transmission electron microscope (Jeol, Japan). Considering the methods used in our previous(8) and current publication, necroptotic EVs were overall purified using either a density-based separation, a size-exclusion method or ultracentrifugation. Purified EVs were then characterized qualitatively and quantitively by NTA, electron microscopy, and flow-cytometry, and for their protein content by western blot and, finally, mass spectrometry. Hence, our results comply with the EV-TRACK consortium requirements of the information necessary to interpret and reproduce EV experiments(9,10).

Figure S2. (Related to Fig. 1) Extracted EVs contain 65 of the 75 most frequently identified proteins in both Exocarta and Vesiclepedia

Venn diagram of total proteins identified in extracted EVs compared with the 100 most frequently identified proteins in Exocarta and and Vesiclepedia.

Figure S3. (Related to Fig. 2) TBQ EVs-enriched proteins share similarity with Exocarta and Vesiclepedia but contain 84 unique proteins

Venn diagram of TBQ EVs-enriched proteins (*e.g.*, the proteins that are significantly upregulated in necroptotic vs. control EVs, with a FDR cutoff of 0.1 and S0 cutoff of 0.1) compared with the exosome proteome data bases, Exocarta and Vesiclepedia.
