# Supplementary figures and images for: Proteomic analysis of necroptotic extracellular vesicles
Source: Cell Death Dis. 2021 Nov 8;12(11):1059. doi: 10.1038/s41419-021-04317-z (PMC8575773; doi:10.1038/s41419-021-04317-z)

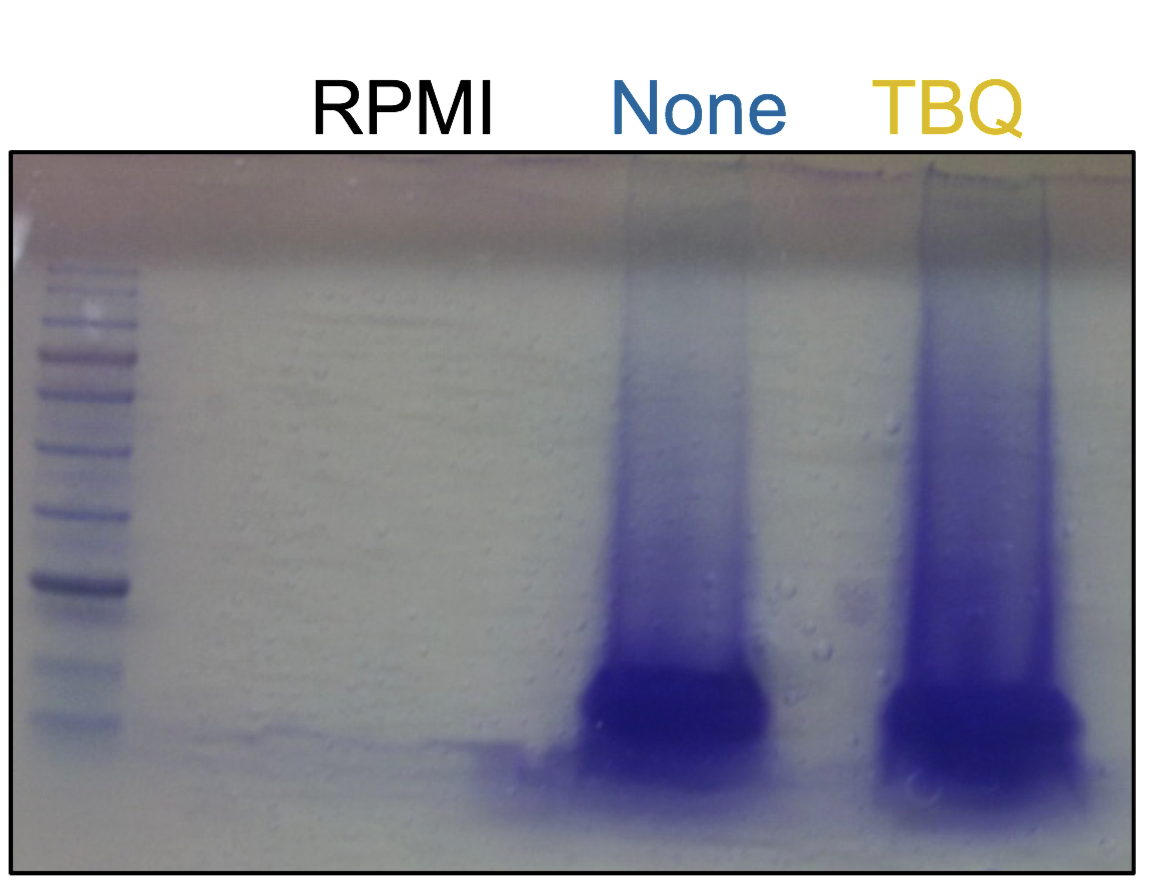

Supplement: Supplementary file 7 — Supplemental Figure 1 [file 41419_2021_4317_MOESM7_ESM.tif]

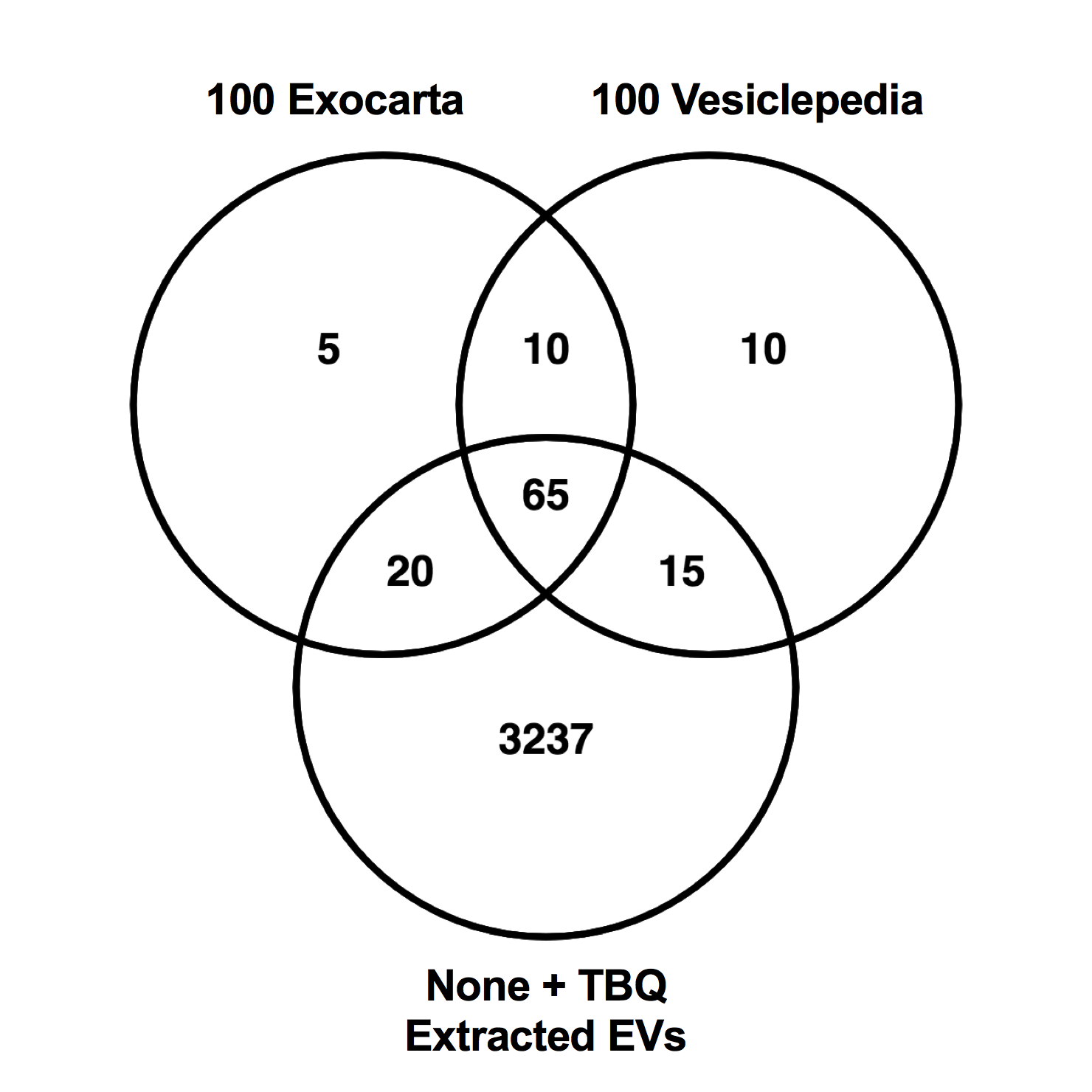

Supplement: Supplementary file 8 — Supplemental Figure 2 [file 41419_2021_4317_MOESM8_ESM.tif]

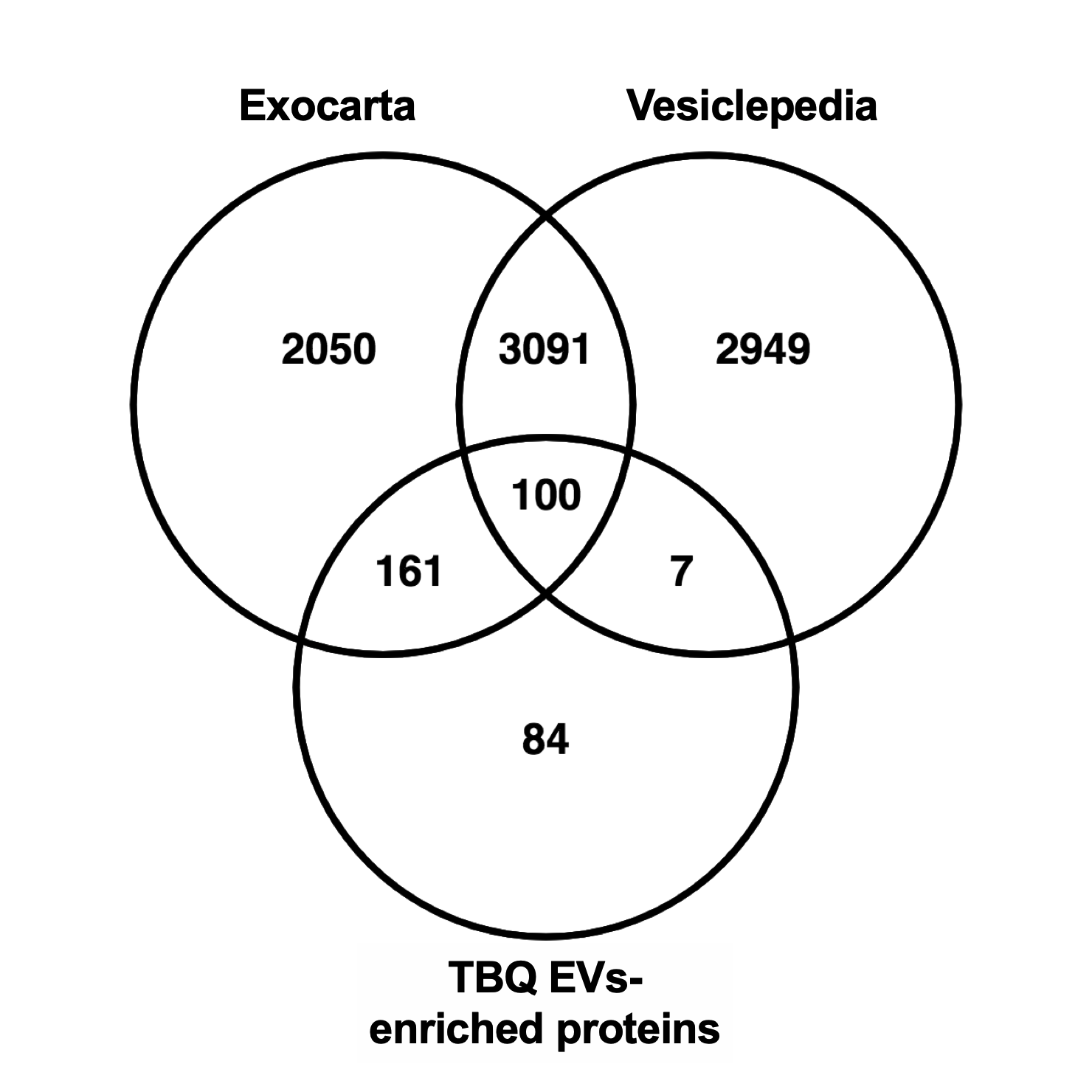

Supplement: Supplementary file 9 — Supplemental Figure 3 [file 41419_2021_4317_MOESM9_ESM.tif]
